# Autism patient-derived SHANK2B^Y29X^ mutation affects the development of ALDH1A1 negative dopamine neuron

**DOI:** 10.1038/s41380-024-02578-6

**Published:** 2024-05-04

**Authors:** Wanjing Lai, Yingying Zhao, Yalan Chen, Zhenzhu Dai, Ruhai Chen, Yimei Niu, Xiaoxia Chen, Shuting Chen, Guanqun Huang, Ziyun Shan, Jiajun Zheng, Yu Hu, Qingpei Chen, Siyi Gong, Sai Kang, Hui Guo, Xiaokuang Ma, Youqiang Song, Kun Xia, Jie Wang, Libing Zhou, Kwok-Fai So, Kai Wang, Shenfeng Qiu, Li Zhang, Jiekai Chen, Lingling Shi

**Affiliations:** 1 Guangdong-Hongkong-Macau CNS Regeneration Institute of Jinan University, Key Laboratory of CNS Regeneration (Jinan University)-Ministry of Education, Guangdong Key Laboratory of Non-human Primate Research, Guangzhou, 510632 China; 2 Center for Cell Lineage and Development, CAS Key Laboratory of Regenerative Biology, Guangdong Provincial Key Laboratory of Stem Cell and Regenerative Medicine, Joint School of Life Sciences, Guangzhou Medical University, Guangzhou Institutes of Biomedicine and Health, Chinese Academy of Sciences, Guangzhou, 510530 China; 3 Centre for Regenerative Medicine and Health, Hong Kong Institute of Science & Innovation, Chinese Academy of Sciences, Hong Kong SAR, 999077 China; 4 University of Chinese Academy of Sciences, Beijing, 100049 China; 5 Raymond G. Perelman Center for Cellular and Molecular Therapeutics, Children’s Hospital of Philadelphia, Philadelphia, PA 19104 USA; 6 Center for Medical Genetics, School of Life Sciences, Central South University, Changsha, Hunan 410008 China; 7 Basic Medical Sciences, University of Arizona College of Medicine-Phoenix, Phoenix, AZ 850004 USA; 8 School of Biomedical Sciences, University of Hong Kong, Hong Kong SAR, China; 9 Department of Psychiatry, the First Affiliated Hospital of Jinan University, Guangzhou, Guangdong 510632 China; 10 Co-innovation Center of Neuro-regeneration, Nantong University, Nantong, Jiangsu 226019 China; 11 Department of Neurology, Hainan General Hospital (Hainan Affiliated Hospital of Hainan Medical University), Haikou, China

**Keywords:** Autism spectrum disorders, Neuroscience

## Abstract

Autism spectrum disorder (ASD) encompasses a range of neurodevelopmental conditions. Different mutations on a single ASD gene contribute to heterogeneity of disease phenotypes, possibly due to functional diversity of generated isoforms. SHANK2, a causative gene in ASD, demonstrates this phenomenon, but there is a scarcity of tools for studying endogenous SHANK2 proteins in an isoform-specific manner. Here, we report a point mutation on SHANK2, which is found in a patient with autism, located on exon of the SHANK2B transcript variant (NM_133266.5), hereby SHANK2B^*Y29X*^. This mutation results in an early stop codon and an aberrant splicing event that impacts SHANK2 transcript variants distinctly. Induced pluripotent stem cells (iPSCs) carrying this mutation, from the patient or isogenic editing, fail to differentiate into functional dopamine (DA) neurons, which can be rescued by genetic correction. Available SMART-Seq single-cell data from human midbrain reveals the abundance of SHANK2B transcript in the ALDH1A1 negative DA neurons. We then show that SHANK2B^*Y29X*^ mutation primarily affects SHANK2B expression and ALDH1A1 negative DA neurons in vitro during early neuronal developmental stage. Mice knocked in with the identical mutation exhibit autistic-like behavior, decreased occupancy of ALDH1A1 negative DA neurons and decreased dopamine release in ventral tegmental area (VTA). Our study provides novel insights on a SHANK2 mutation derived from autism patient and highlights SHANK2B significance in ALDH1A1 negative DA neuron.

## Introduction

SHANKs have emerged as prominent candidates associated with neurodevelopmental disorders, such as ASD and intellectual disability [1–3]. Among the SHANKs family, SHANK2 gene is the largest and encodes large scaffolding proteins. These proteins are enriched in the postsynaptic density (PSD) of excitatory synapses and possess multiple functional domains for protein-protein interaction. Notably, SHANK2 undergoes complex transcriptional regulatory events, with multiple intragenic promoters and alternatively splicing exons, resulting in the expression of isoforms that are specific to particular spatiotemporal contexts [4, 5]. So far, mounting evidence has established a link between SHANK2 and neurodevelopmental disorders by overexpressing SHANK2 mutants in primary culture neurons [6, 7], utilizing iPSCs carrying patient-derived SHANK2 mutants [8–10] or employing gene-edited SHANK2 knockdown models [11]. These gain-of-function and loss-of-function studies have provided insights into the molecular function of SHANK2 and their impacts on electrophysiological and behavioral outcomes. However, these studies have failed to capture the broad spectrum of phenotypes associated with various mutations observed in SHANK2 [2, 7, 8, 12, 13]. One bottleneck is the lack of the characterization of each isoform with spatial and functional specificity.

To address this critical gap, we have collected an autism pedigree carrying a single point genetic mutation of SHANK2B. By intergrating characterization of this mutation using in vitro iPSCs and in vivo mouse model, we have uncovered its impacts on a subtype of dopamine neuron during early developmental stage. This study sheds light on the vital role of diverse transcripts in autism and introduceds a new paradigm for unraveling the complex mechanism underlying heterogeneity of ASD.

## Methods

### Cell source

We generated iPSCs from the peripheral blood mononuclear cells (PBMCs) of an ASD pedigree and control individuals. Specifically, we used two clones (PC1, PC2) derived from the male ASD patient, two clones (HC1, HC2) from his unaffected father, and two clones (Ctrl1, Ctrl2) from a healthy male individual who is not related to the ASD pedigree.

### Mutation identification

Whole genome sequencing (WGS) was performed to screen for any autism-related mutation in autism patient in comparison with his unaffected parents. Based on a previous sequencing panel [14], our analysis revealed a single de novo heterogenous stop-gain mutation Y29X (C to G) in SHANK2 gene (NM_133266.5) and no other stop-gain mutations or frameshift indels were identified upon manual inspection. Furthermore, no other de novo SNV mutations were found to be related to ASD.

### Sanger sequencing

Cell suspension was prepared by digesting the iPSCs with EDTA and centrifuged at 12000 rpm for 1 min. The supernatant was discarded, and 450 μl lysis buffer (1 M Tris-HCl, 0.5 M EDTA, 5 M NaCl, 10% SDS) with 50 μl proteinase K (1 mg/ml, Merck, 39450-01-6) were added. The cells were then lysed at 55 °C for 2 h. Extracted DNA were sent to TIANYIHUIYUAN Company for Sanger sequencing. The genotypes of iPSCs cell lines were identified through the DNA sequencing analysis (Chromas).

### Genetic editing technology

To establish the Iso1 cell line model, SHANK2 point mutation Y29X (C to G) was edited by Biocytogen Extreme Genome Editing System (EGE) based on CRISPR/Cas9 and developed by Biocytogen. The candidate sgRNAs, located in the SHANK2 intron1, were screened by the CRISPR design tool (http://www.sanger.ac.uk/htgt/wge/). The selected RNAs with high specificity were then determined according to on-target activity using UCATM (Universal CRISPR Activity Assay). The sgRNA-mediated CRISPR/Cas9 system-induced Nuclease-induced double-strand breaks (DSBs) can be homology-directed repair (HDR) pathway when endogenous donor DNA with homologous arms is present. The donor plasmid containing the 5′ (1.3 KB) and 3′ (0.9 KB) homologous arms, as well as the insertion of gene fragment with the point mutation c. 87G>C and a resistance cassette (puroDeltatk) were co-electoporated into the Ctrl1 cells to establish Iso1 cell line.

Both PC and Iso1 cell line were heterozygous, carrying Y29X (C to G) mutation in one allele. Only PC1-res cell line was homozygous, as the Y29X mutation had been repaired (G to C).

### Cell culture

#### iPSCs culture

Neuronal induction and differentiation of iPSCs clones was performed according to our previous protocol [11, 15]. Briefly, cells were cultured in six-well plates coated with Matrigel (BD Matrigel^TM^, hESC-qualified Matrix, 354277), and grown in mTeSR^TM^ medium (Stemcell Technologies, 85850). The cells were digested using EDTA (5 × 10^−4^ mol/L, Thermo Fisher Scientific, 25200056) and passaged manually, every 3–4 days for 2–3 times. OCT4 and SSEA4 were used to assess the pluripotency of cells.

#### Neuronal induction

Neural progenitor cells (NPCs) were generated through a neuronal induction process lasting for more than 15 days, starting from iPSCs. NPCs were derived using a dual SMAD and Wnt inhibition method, with manual isolation of neuronal rosettes. During the initial 8 days of differentiation, the cells were cultured in the N2B27 medium (Thermo Fisher Scientific, 17502048, 17504044) supplemented with SB431542 (5 µmol/L, Selleck, S1067) and dorsomorphin (5 µmol/L, Selleck, S7840), referred to as the SMAD neural proliferation system I. The medium was changed every 2 days. After 8 days, neural rosettes were carefully scraped from the culture surface and transferred onto a Matrigel-coated plate. They were then maintained in N2B27 medium for an additional 8 days. On the 16th day of proliferation, rosettes were mechanically scraped into floating fragments and plated into non-coated T25 flask for floating culture. The neural proliferation system II was used, which consisted of N2B27 medium supplemented with 20 ng/mL bFGF (Thermo Fisher Scientific, PHG0266) and 20 ng/mL EGF (Thermo Fisher Scientific, PHG0315). To expand NPCs, they were dissociated with Accutase (StemPro Accutase cell dissociation reagent, Thermo Fisher Scientific, A1110501) and passaged for 3 times. NPCs were characterized by expression of NPCs marker NESTIN and SOX2, which are indicative of their neural progenitor identity.

#### Neuronal differentiation

For neuronal differentiation, NPCs were expanded for 3 passages and then dissociated into single cells. These cells were plated directly on glass coverslips coated with a glial cell feeder layer which was prepared from neonatal mouse astrocyte (P0–P3), at a density of 10^5^ cells per well in 24-well plates for molecular assays. The cells were then fed with a neuronal differentiation medium (N2B27) supplemented with 1 µM Dibutyryl cyclic-AMP (Sigma, S7858) and 20 ng/μL BDNF (PeproTech, 450-02-10). The medium was changed every other day. Following that, the cells were collected from the coverslips for immunocytochemistry. The differentiated cells were positive for various neuronal markers, including tyrosine hydroxylase (TH), vesicular glutamate transporter 1 (VGLUT1), and vesicular GABA transporter (VGAT). These results indicate that the NPCs were capable of differentiating into various types of neurons.

#### Neuron induction with SHH

The iPSCs were cultured as described above. When the confluence of iPSCs reached 95%, the cells were detached with EDTA (5×10^−4 ^mol/L) and replated into 12-well plate. After 1–2 days, when the cells reached full confluence, the medium was changed to neural proliferation system I to initiate neuronal differentiation. Following 8 days of neuronal induction, cells were mechanically scraped into floating fragments and replanted into two wells of a 6-well Matrigel-coated plate. The cells were then cultured in N2B27 medium supplemented with sonic hedgehog (SHH, 100 ng/mL, R&D system, 464-SH-025) and fibroblast growth factor-8 (FGF8, 100 ng/mL, R&D system, 423-F8-025). On the 8^th^ day of proliferation, cell clones from one well of the 6-well plate were mechanically scraped into floating fragments and then replated into non-coated T25 flask for floating culture with neural proliferation system III (N2B27 + 20 ng/mL bFGF + 20 ng/mL EGF + SHH + FGF8). Typically, neurospheres usually formed on the second day of culture in neural proliferation system III. Following formation of neurospheres, Accutase was then used to detach them, and the single cells were plated in Matrigel-coated dishes with neuronal differentiation medium (N2, Thermo Fisher Scientific; B27, Thermo Fisher Scientific; 1 µM dibutyryl-cAMP, Sigma; 20 ng BDNF, PeproTech; 20 ng/mL GDNF; 200 mM AA).

#### Virus transduction

NPCs were digested into single cells and infected with lentivirus (CAMKII, LV-gene (44213-1), Genecham, GOSL0188572, 6 × 10^8^ TU/mL) packed with a tetracycline-controlled red fluorescent protein (RFP) expression construct. This allowed for visualization of fluorescent images and subsequent morphological reconstruction of glutamertagic neurons. Additionally, NPCs were digested into single cells and infected with lentivirus (expressed under the control of DAT promoter) to label dopaminergic neurons for electrophysiological recordings. After 6 h of infection, the cells were centrifuged, the medium was changed. The following day, the cells exhibiting RFP signals were cultured on coverslips coated with a glial cell feeder layer at a density of 6 × 10^4^ cells per well in a 24-well plate to promote neuronal development.

#### Immunocytochemistry (ICC)

Cells grown on coverslips on 9th day of neural induction from NPCs were fixed using 4% paraformaldehyde (PFA) for 15 min, washed 3 times for 5 min each with PBS, permeabilized with PBS containing 0.3% Tween-20 (Solarbio, T8220) (PBST), and blocked by PBST containing 3% bovine serum albumin (BSA, VETEC^TM^) at room temperature (25 ± 1 °C) for 1 h. After blocking, the cells were incubated with the primary antibodies (Table S1) diluted in PBST containing 1% BSA overnight at 4 °C. The next day, cells were washed 3 times and 10 min each with PBS and incubated with secondary antibodies diluted in PBS at room temperature for 1 h. To analyze the neuronal morphology, images of neurons were acquired using 20×, 40× objective (Imager Z2, Zeiss) and digitized them using a Zeiss camera (Axiocam 506 mono, Zeiss), axon, dendrite, and soma were traced, and morphology, including dendrite length and number of dendrite branches were analyzed using neurolucida 360 for Sholl analysis.

#### Quantitative real-time PCR (qRT-PCR)

mRNA extracted from neurons harvested on 9th day of neural induction from NPCs or mouse brain tissues were converted to cDNA using PrimeScript RT Reagent Kit with gDNA Eraser (#RR047A, Takara). SHANK2E and SHANK2A transcripts were produced from primer “Human long SHANK2 transcripts”. Primer “Mouse Shank2A” was designed to target exon 1 and 2 of Shank2A (NM_001113373.3), primer “Mouse Shank2B” was designed to target exon 1 and 2 of Shank2B (NM_001081370.3), primer “Mouse Shank2E” was designed to target exon 9 and 10 of Shank2E (XP_006508583.1). Primers used were listed in supplementary table (Table S2). qPCR reactions were performed using TB Green® Premix Ex Taq™ II (Tli RNaseH Plus) (#RR820A, Takara) in triplicates for each sample. GAPDH was chosen as reference gene. Initial denaturation was 95 °C for 30 s, the amplification cycles were 95 °C for 5 s, 60 °C for 30 s, for 40 repeats. All qRT-PCR reactions were performed and analyzed using the StepOne Plus Real-Time PCR System (Applied Biosystems) with the comparative Cq method.

#### Western blot assay

Proteins were extracted from neurons harvested on 9th day of neural induction from NPCs or brain tissues from 8-week-old mice by adding appropriate amount of RIPA lysis buffer (Thermo Fisher Scientific, 89900) containing 1% protease inhibitor (Merck, 539137-10VL). After sonication and centrifugation, extracted protein were obtained for analysis. Protein concentrations were measured using Pierce^TM^ BCA Protein Assay Kit (Thermo Fisher Scientific, 23209). Protein were mixed with 5× loading buffer (Beyotime, P0015) and heated at 95 °C for 8 min, then separated on SDS-PAGE gel (Beyotime, P0012A) and transferred to a polyvinylidene difluoride (PVDF) membrane (Millipore, IPVH00010). Membranes were blocked in TBS with 0.2% Tween-20 (TBST) (Solarbio, T8220) containing 5% bovine serum antigen (BSA) at room temperature for 1.5 h and incubated with primary antibody (Table. S1) at 4 °C overnight. On the following day, the membranes were rinsed by TBST for 5 times for 8 min each and incubated with anti-Rabbit IgG, HRP-linked antibody (1:4000, 7074P2, Cell Signaling) at room temperature for 1.5 h, then rinsed 5 times for 8 min again. Protein signals were developed using ChemiDoc Touch Imaging System (Bio-RAD, USA).

#### Electrophysiological recordings

For electrophysiological recordings, iPSCs-derived neurons were used after 4-5 weeks of culture. Patch-clamp recordings were performed using an IR-DIC microscope (Nikon Eclipse FN-1 microscope) and an amplifier (MultiClamp 700B, Molecular Devices). Cells were incubated at room temperature in the artificial cerebrospinal fluid (ACSF, in mM: 126 NaCl, 26 NaHCO_3_, 10 D-glucose, 2.5 KCl, 2 CaCl_2_, 2 MgCl_2,_ and 1.25 NaH_2_PO_4_, pH 7.4) that was oxygenated with 95% O_2_ and 5% CO_2_. Borosilicate microelectrodes with a resistance of 4-8 MΩ were pulled using a pipette puller (Narishige PC10). For recordings on intrinsic membrane properties, the pipettes were filled with a solution (pH 7.3) containing: 4 mM KCl, 126 mM K-gluconate, 10 mM HEPES, 0.3 mM Na_2_-ATP, 4 mM Mg-GTP, 10 mM phosphocreatine. Cell slide was placed in a recording tank containing ACSF with oxygen supplementation. Neurons were visualized with a 40x water objective on an IR-DIC microscope and recorded using the amplifier. The recorded signals were filtered with a low pass frequency of 3 kHz and digitized at a frequency of 20 kHz (DigiData 1550 A, Molecular Devices). In the current-clamp mode, the input resistance (Rin) and positive resting membrane potential (RMP) were recorded. Neurons were subjected to positive and negative current stimuli of −10 pA and 2 pA per second, respectively, and the slope of the linear fit of the current-voltage curve was used to determine the input resistance (Rin). The RMP was recorded as the uncorrected fluid-contact potential. The neurons were then recorded in voltage-clamp mode, in which voltage was maintained at −70 mV. A sodium current was induced by increasing the voltage by 5 mV every 300 ms from −20 to 50 mV, and the peak value and threshold of sodium current were recorded. The neurons were then recorded in current-clamp mode. The current was increased by 10 pA every 1000 ms from −30 pA to 80 pA, and the peak and frequency of the action potential were recorded. Spontaneous excitatory postsynaptic current (sEPSC) and spontaneous inhibitory postsynaptic current (sIPSC) were recorded at −70 mV and 0 mV potentials, respectively. The collected electrophysiology data were analyzed with pClamp10 (Molecular Devices).

#### Bulk RNA-sequencing

We conducted an analysis of the transcriptome of neurons derived from PC1 and HC1 clone on 9th day of neural induction from NPCs. To ensure data quality, we utilized cutadapt (version 2.6) for the processing of raw reads. This involved the removal of adapter sequences and elimination of low-quality reads. The resulting RNA-seq clean reads were then aligned to the human reference genome (GRCh38) using STAR (version 2.7.10a). For gene expression quantification, we employed RSEM (v1.2.28) with GENCODE annotation (human release 31). Specifically, we extracted the Transcripts Per Million (TPM) values of the SHANK2 gene, as well as its SHANK2E, SHANK2A, and SHANK2B transcripts, from the RSEM results. Finally, we utilized the ggplot2 package to visualize the obtained data.

#### BaseScope

Probe design was conducted by Bio ACD company. SHANK2E: length: 1849 amino acid (aa), targeting 496-716 of NM_012309.4; SHANK2A: length: 1470 aa, targeting 36-111 of ENST00000656230.1; SHANK2B: 1261 aa, targeting 2–78 of NM_133266.5. For assessing the expression of various transcripts, HC1, PC1 and PC1-res1 NPCs were digested into single cells. Cells were inoculated onto a chamber culture slide. After 5 days culture, the neurons were pretreated according to protocol (Advanced Cell Diagnostics, 322381). For SHANK2A expression, iPSCs from HC1 and PC1 were inoculated onto a chamber culture slide 1 day before further treatment. Briefly, cells were gradiently dehydrated and rehydration on slides before being treated with H_2_O_2_ and protease III from RNAscope® H_2_O_2_ and Protease Reagents (Advanced Cell Diagnostics, 322381) at room temperature for 10 min. Probe hybridization and signal amplification were performed according to the manufacturer’s instructions. Briefly, cells were separately detected the fast red detection of C2 signal and green detection of the C1 signal, using the BaseScope^TM^ Duplex Detection Reagents (Advanced Cell Diagnostics, 323800). Subsequently, the same slides were hybridized and combined with TH (Millipore, AB152, 1:1000) immunohistochemistry (IHC) staining. The secondary antibody, donkey anti-rabbit Alexa-488 (Invitrogen^TM^, A21206, 1:500) was used. Finally, the slides were dried at 60 °C for 15 min and mounted using VectaMount (Vector Labs Vectamount, 321584). The probe signals were detected under standard bright field (Zeiss).

#### Generation of Shank2 KI mice

All animal experimental protocols have been approved by the Ethics Committee of Experimental Animals of Jinan University in accordance with IACUC guidelines for animal research. The sample size was determined based on literatures from the same field, and in consideration of animal size limit due to animal welfare requirement and animal experimental ethical code. Randomization methods were not used in this study.

The 29th amino acid (Y29; codons TAC) in the human SHANK2 gene corresponds to the conserved 28th amino acid (Y28; codons TAC) in the mouse SHANK2 gene. The 28th amino acid residue Tyr was located within the exon 16 (ENSMUSE00000756726) of the mouse SHANK2 gene. This design was based on mouse SHANK2 transcript-003 (NM_001081370) and completed by Biocytogen. Using CRISPR/Cas9 technology, human-carried SHANK2B^*Y29X*^ mutation was introduced into C57BL/6 mice, which replaces the codon encoding Tyr with a stop condon to obtain heterozygous SHANK2 (SHANK2B^*Y29X*^) mice. At least 5 times backcrossing were done to remove off-target effects brought by CRISPR procedure. Subsequently, heterozygous mice were interbred to obtain homozygous (SHANK2 KI) mice. If not otherwise indicated, behavioral and molecular assays were performed on male wild-type (WT) and homozygous SHANK2 KI mice at 8-9 weeks of age.

#### Genotype identification

For genotype identification, gingeli size ear or tail tissue of mice was obtained 3 weeks after birth. 300 μl lysis buffer (1 M Tris-HCl, 0.5 M EDTA, 5 M NaCl, 10% SDS) containing 6 μl proteinase K (Merck, 39450-01-6) was added to the tissues and then transferred to a 55 °C oven overnight in order to digest the tissue completely. DNA was extracted by phenol chloroform (Solarbio, P1013) and purified by absolute ethanol then 70% ethanol. Extracted DNA was dried at 37 °C overnight and then dissolved by 200 μl ddH_2_O and stored at 4 °C until use. In the Hot Start enzyme PCR reaction program, DNA template, two primers (Primer F: 5′-TGCCTGGATCTGAGCTGGGTGATTA-3′; Primer R: 5′-CATGCCTGCAGGAAGCCTAGATGTT-3′), Taq enzyme (TAKARA, RR003A), ddH_2_O, were mixed and followed by the PCR program (Biometra, Germany). DNA samples were sent to TIANYIHUIYUAN Company for Sanger sequencing. The genotypes of mice were identified using the DNA sequencing analysis software (Chromas).

#### DAB staining

Mice were anesthetized with isoflurane and perfused with PBS, followed by 4% PFA. The brain was isolated and post-fixed in PFA solution overnight, and gradiently dehydrated in 20% and 30% sucrose at 4 °C. Brain slides in 40 μm thickness were obtained using a freezing microtome (CM1950, Leica, Germany). After being rinsed by PBS for 3 times and 10 min each, brain slides were immersed in H_2_O_2_ for 30 min, followed by another 3 times rising by PBS. Slides were then blocked in 5 ml PBS containing 5% BSA and 0.5% normal goat serum (Sigma, 566380) (blocking buffer) at room temperature for 1 h. The primary antibody (anti-TH, Millipore, AB152, 1:300) diluted in blocking buffer was added, and incubated at 4 °C for 24 h. After being rinsed by PBS for 3 times, the slides were incubated with the secondary antibody coupled to peroxidase (1:500) at 4 °C overnight, followed by 3 times rising by PBS. The DAB staining solution was prepared according to DAB staining kit (Cell signaling, 8059S). Brain slices were immersed in 2 ml DAB solution until appropriate staining was observed under an optical microscope and DAB solution was replaced by PBS to terminate staining. After air dried, brain slides were gradiently dehydrated with 70% ethanol, 95% ethanol, absolute ethanol, each for 10 min and finally with xylene. Once removed from xylene, slides were mounted, completely dried and stored at room temperature until use.

#### Immunofluorescence (IF) staining

Brain slides in 30 μm thickness were obtained for IF staining. After rinsed by PBS for 3 times, slides were blocked in PBS containing 0.3% TritonX-100 and 5% donkey serum (Solarbio, SL050) at room temperature for 2 h. Slides were then incubated with the primary antibodies (Table S1) diluted in PBS at 4 °C for 24 h. After being rinsed by PBS for 3 times, sliced were incubated with the secondary antibody diluted in PBS at room temperature for 1.5 h. Slices were then rinsed in PBS, mounted, covered and visualized with laser scanning confocal microscope with ×20, ×40, ×63 magnification equipped with 405/488/546/647 nm lasers (Axiocam 506 mono, Zeiss, Germany). For mean fluorescence intensity analysis of TH on NAc brain region, five 250μm × 250μm subregions from one image were obtained to get an average of fluorescence intensity for one image field. *n* ≥ 10 image fields from 3 WT mice and 3 KI mice were included in analysis. The intensity of TH was normalized to the average of WT group for all analyzed brain regions.

#### Open field test

Mice were placed in the central area of the apparatus (50 × 50 × 40 cm, Noldus, Netherlands) at the beginning and allowed to explore the arena for 15 min.

#### Elevated plus-maze

The elevated plus-maze (Noldus, Netherlands) is consisted of a central area, two open arms, and two closed arms, which is 50 cm height to the ground. Mice were placed in the central area heading to the open arm at the beginning of the assay and allowed to explore the arena for 5 min.

#### Morris water maze test

In the Morris water maze test, mice underwent a training trial for five consecutive days. During each training session, mice were trained four times a day to locate a hidden platform (10 × 10 cm) in the circular apparatus (120 cm in diameter) with visual cues on the wall. If the test mouse failed to find the platform within 60 s, it was guided to the platform and allowed to stay on it for 10 s. Escape latency to platform was recorded once the mouse found and stayed on the platform for 10 s. On the 6th day, a probe trial was conducted in which the platform was removed, and test mouse was allowed to explore the arena for 1 min. Escape latency to platform, time spent in target quadrant (where the platform was originally locate) were recorded and used to assess learning and memory function.

#### Social interaction assay and repetitive behavior in home cages

For the social interaction assay and repetitive behavior recording, a plastic apparatus (15 × 8 × 12 cm) with beddings was used as the home cage. Test mice were habituated in the plastic apparatus for 2 days before the assay. In the beginning of the assay, the empty cylindrical apparatus (5 cm in diameter) was placed at one side of the home cage. In the first phase of the social interaction assay, a test mouse was allowed to freely move around the home cage and explore the cylinder for 10 min. In the second phase, a stranger mouse of the same gender and smaller in size as the test mouse was placed into the cylinder, and the test mouse was allowed to explore the stranger mouse for 10 min. The duration spent on sniffing on the cylinder in each phase were recorded to demonstrate the social preference. For repetitive behavior, mouse was allowed to freely explore the home cage for 5 min, the duration spent on jumping, grooming and digging were manually recorded by one experimenter.

#### Three-chamber social assay

The three-chamber social assay takes place in a transparent apparatus (62 cm × 40 cm × 40 cm, Noldus, Netherlands) that is divided into three chambers (left, middle, right) by thin transparent wall, with dim light in the behavioral room. In the first phase, inanimate objects (O1 and O) was placed in two small cylindrical containers in the left and right chamber, respectively. The test mouse was initially placed in the middle chamber and allowed to freely explore all three chambers for 10 min to acclimate to the environment. In the second phase, the test mouse was gently guided back to the center chamber, and the two entrances to the side chambers were blocked. One of the containers (O1) was replaced with a strange mouse (Stranger 1, S1), while the other container retained the inanimate object (O). Then, the two entrances were opened to allow the mouse to explore the chamerbs for 10 min. In the third phase, the test mouse was gently guided back to the center chamber again, with the entrances to the side chambers blocked. The remaining container with the inanimate object (O) was replaced with Stranger 2 (S2), representing an unfamiliar novel mouse. The test mouse was allowed to explore and interact with either Stranger 1 or 2 for 10 min. The duration and frequency of sniffing on the containers were manually calculated by two experimenters. Preference ratio is calculated by sniffing duration (S1 − O)/(S1 + O) in second phase, and (S2 − S1)/(S2 + S1) in third phase, to represent social preference and social memory recognition, respectively.

#### Rotarod test

The Rotarod apparatus (47700(rat)/600(mouse)) was used in the test. As the test started, the rod accelerated from 0 rpm to 80 rpm in 5 min. The standard motor learning task was performed as three trials per day for three consecutive days. Mice were allowed to take a 5 min rest between trial. The duration that each mouse stays on the rotating rod in each trial was recorded as the latency to fall.

#### In vivo fastscan cyclic voltammetry (FSCV)

FSCV was used to measure dopamine fluctuations in the nucleus accumbens (NAc). Mice were anesthetized with urethane (1.5 g/kg, i.p.) and placed in a stereotaxic frame equipped with a heating pad to maintain body temperature (Harvard Apparatus, Holliston, MA, USA). A bipolar, stainless-steel stimulating electrode, coupled with a 26-gauge infusion cannula (Plastics One, Roanoke, VA, USA), was placed into the VTA (AP − 5.2 mm, ML + 0.5 to −1.5 mm, DV between −7.4 and −8.1 mm), a carbon fiber microelectrode was implanted in the NAc core (AP  + 1.2 mm, ML − 1.4 mm, DV from −6.0 to 7.0 mm) and an Ag/Ag Cl reference electrode was placed in the contralateral hemisphere. A low-pass filtered (2 kHz) triangular waveform (−0.4 to +1.3 V and back to −0.4 V at a rate of 400 V/s, repeated at 100 ms intervals) was applied to the carbon fiber microelectrode. Data were digitized and processed using NI-6711 and NI-6251 DAC/ADC cards (National Instruments, Austin, TX, USA) and Demon Voltammetry and Analysis Software (Wake Forest Baptist Medical Center, Winston-Salem, NC). Background-subtracted cyclic voltammograms (CVs) were obtained by digitally subtracting stable background currents to resolve CVs for dopamine. Electrical pulses delivered to the VTA were computer-generated with a 6711 PCI card (National Instruments, Austin, TX, USA) and were optically isolated from the electrochemical system (NL 800 A, Neurolog, Digitimer Ltd, Hertfordshire, UK). Electrical stimulation for phasic evoked DA release consisted of biphasic square wave pulses (300 μA, 24 pulses, with a 4 ms pulse width, applied at 60 Hz). For tonic-evoked DA release, all parameters remained the same except that the frequency was changed to 5 Hz. VTA stimulation, and subsequent dopamine current recordings detected by electrode in the NAc, were performed with a 3 min interval between each stimulation to allow releasable DA stores to baseline. For the FSCV experiment, at baseline, 6 evoked-DA samples were first collected.

#### Whole-cell patch clamp recording

Mice were sacrificed at postnatal 38–40 days, coronal slices containing the VTA were used for recording miniature excitatory postsynaptic current (mEPSC). Slices were immersed in ice-cold ACSF saturated with 95% O_2_ and 5% CO_2_ and incubated at 32 °C for 30 min before being kept at room temperature. The slices were then switched to the recording chamber circulated with ACSF. The recording electrode contains a potassium-based solution (in mM: 130 K-gluconate, 4 KCl, 2 NaCl, 10 HEPES, 4 ATP-Mg, 0.3 GTP-Na, 1 EGTA, and 14 phosphocreatine, pH 7.2, 295 mOsm), and has an electrical resistance of 4 ~ 6 MΩ. Neurons were voltage-clamped at −70 mV. VTA neurons were patch clamped with an electrode containing a potassium-based internal solution that contains 0.25% biocytin.

#### Morphological reconstruction and analysis of VTA TH+ neurons

When the whole-cell patch clamp recordings were completed, the cell received 500 pA of current to allow the exchange of the cell fluid and electrode fluid, and biotin in the electrode fluid was exchanged into the cell for cell morphology construction. ACSF was discarded, the recorded brain slices were transferred to 24-well plates containing 4% PFA at 4 °C overnight, then rinsed by PBS for 3 times and 10 min for each. The brain slices were blocked in blocking buffer (5% donkey serum and 5% goat serum in PBS containing 0.3% TritonX-100) at room temperature for 1 h and stained with the primary antibody diluted in blocking buffer at 4 °C overnight. After 3 times rinse by PBS for 10 min each, slices were incubated in mixed secondary antibody, including both sheep-rabbit immunofluorescence secondary antibody and anti-biotin avidin coupled secondary antibody, at 4 °C overnight, followed by rinsed by PBS. Brain slices were visualized under laser scanning confocal microscope and dendritic spines of TH positive dopamine neurons with biotin staining were analyzed. Mushroom and stubyby protrusion from the dendritic branch were identified as spine. At least three dendrite segments in each neuron were included in analysis, each analyzed dot represented one neuron. Data came from more than 8 mice in each group.

#### Single Cell RNA-Seq (scRNA-Seq)

WT and KI mouse midbrain tissues were obtained on embryonic day 13.5, and subjected to single-cell RNA-Seq. To achieve a homogenous single-cell suspension, the Worthington Papain Dissociation System kit (Worthington, LK003153) was used following the manufacturer’s recommended protocol. Briefly, the midbrain tissues were washed with DMEM/F12 and then dissected into small pieces in Papain/DNase solution. The tissue suspension was subsequently transferred to an orbital shaker set at 60 rpm/min and maintained at 37 °C for 30 min. Gentle trituration was performed to obtain a single-cell suspension, and the papain was inactivated using an albumin-ovomucoid inhibitor solution. Cells were pelleted at 300 g, resuspended in PBS containing 0.04% BSA (Sigma, A2153), and adjusted to a concentration of 500–1000 cells/μl.

For the scRNA-Seq, a 3′-library was constructed utilizing the Chromium Single Cell Reagent Kits v3 (10X GENOMICS) according to the manufacturer’s user guide. The amplified and purified library was quantified using the Quant-iT dsDNA Assay Kit, high sensitivity (Thermo Fisher) on Qubit 2.0, and subsequently sequenced with PE 150 (paired-end sequencing, 150 bp reads).

To align the clean reads obtained from the scRNA-Seq to the mouse reference genome (mm10), the STAR (v.2.7) software was employed. The resulting BAM files were sorted and indexed using the sort and index functions provided by SAMtools (v1.9). To visualize the aligned reads, the BAM files were viewed using the Integrative Genomics Viewer (IGV).

#### SMART-Seq single-cell data analysis

To assess expression of SHANK2 isoform in the midbrain, we re-analyzed SMART-Seq single-cell data of the human ventral midbrain from La Manno et al. [16]. First, the single cell RNA-seq reads were aligned to the human reference genome (hg38) using Hisat2 (version 2.1.0). The output of hisat2 is a SAM file. Then we transformed from SAM files to BAM files and sort the BAM files using Samtools (version 1.9). Quantification of isoform expression was performed using StringTie (v1.3.3b) with GENCODE annotation (human release 31). We extracted read count information directly from the files generated by StringTie using prepDE.py mentioned in the Stringtie manual. The count value of Transcript for each cell was normalized using CPM (Counts Per Million) and then log transformed.

We acquired processed data including the gene counts matrix and cell meta data. UMI counts were normalized for each cell by the total expression, multiplied by 10,000 scaling factors, and log-transformed. The highly-variable genes (HVGs) were identified using the function highly_variable_genes with min_mean = 0.0125, max_mean = 3, min_disp = 0.5. The scale function was used to regress out variation due to differences in total UMIs per cell. We chose terminally differentiated cell types (hDA1, hDA, hEndo, hGaba, hMgl, hOMTN, hOPC, hPeric, hSert) for analysis. To reduce noise and the gene expression space further, we then performed principal component analysis (PCA) on the scaled data for the variable genes. The neighborhood graph of cells was computed using the top 30 PCA vectors space, and the number of neighboring data points was set to 10. t-SNE (t-distributed Stochastic Neighbor Embedding) was used for visualization.

Finally, we employed heatmaps to visualize the expression of marker genes across different cell types, as well as the three transcripts of SHANK2: SHANK2A, SHANK2B and SHANK2E within each cell type. Gene expression values and isoform expression values were standardized between 0 and 1.

### Statistical analysis

Except RNA-Seq, all statistical analyses were performed using GraphPad Prism8 (USA). All data presented in the study are mean ± standard error (SEM) unless otherwise noted. No data were excluded from this study. Data were firstly tested for normality. If not otherwise indicated, parametric data were compared using unpaired Student’s *t*-test, Two-way ANOVA followed by Šídák’s multiple comparisons test, or One-way ANOVA followed by Tukey’s post-hoc comparison test. Non-parametric data were compared using Mann–Whitney test and Wilcoxon test. All reported p values are two-sided. All parametric tests were made based on the non-equal variance assumption. p < 0.05 was regarded as significance. BaseScope on cell line, behavioral tests and molecular assays on animals were analyzed by persons who were blinded to the grouping. Other analysis were analyzed by persons who were not blinded to the grouping.

## Results

### SHANK2B^*Y29X*^ carrier shows defects in DA neurons differentiation, dendritic morphology and function

**Fig. 1:**
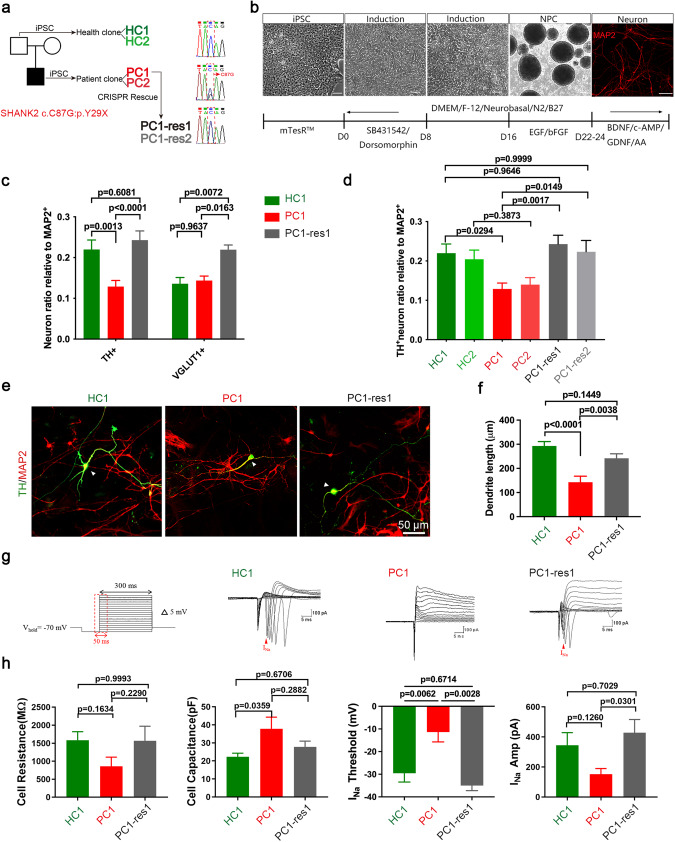
The SHANK2B^*Y29X*^ mutation from the ASD proband affects the early development of DA neurons, which can be partially rescued by genetic editing. **a** iPSCs were generated from an ASD pedigree. Two clones were derived from the unaffected father (HC), giving HC1, HC2. Two clones were derived from the autism proband (PC) carrying heterogenous C87G mutation on SHANK2 gene, giving PC1, PC2. CRISPR mediated genetic rescuing G to C on one of the proband’s iPSCs (PC1) and derived to two clones, PC1-res1 and PC1-res2. **b** Schematic representation of a modified dual-SMADi monolayer differentiation protocol for the induction of iPSCs into NPCs as observed under bright field. NPCs were seeded in separate wells and differentiated into neurons, as assessed by ICC staining of the neuronal marker MAP2 (red). Scale bar = 50 μm. **c** Differentiation potential of dopamine neurons and glutamatergic neurons was determined by the proportion of TH-positive cells and VGLUT1-positive cells normalized to MAP2-positive cells, respectively (*n* ≥ 10 image fields from each clone were analyzed). **d** Differentiation potential of dopamine neurons, determined by the proportion of TH-positive cells normalized to MAP2-positive cells, was decreased in PC clones compared with HC clones. This differentiation defect could be rescued by genetic correction, as observed in PC1-res clones. (*n* ≥ 10 image fields from each clone were analyzed). **e** Immunostaining of TH (green) and MAP2 (red) in neurons harvested 9 days differentiation from NPCs. Arrowhead indicates a DA neuron co-expressing TH and MAP2. Scale bar = 50 μm. **f** Dendritic length of TH positive DA neurons was decreased in PC1, while genetic correction could rescue this dendritic morphological defect as observed in PC1-res1 cells. Neuron reconstruction based on MAP2 signal was performed using neurolucida 360 and analyzed by Sholl analysis. (*n* = 27 TH + MAP2+ cells in HC1, 19 in PC1, 28 in PC1-res1 clone). **g**, **h**, Electrophysiological property (Sodium channel functioning) of NPCs-derived DA neurons after more than one month of culture (*n* = 14 cells in HC1, 13 in PC1 and 10 in PC1-res1 clone). Data are represented as the mean ± SEM. Statistical significance was evaluated by One-way ANOVA, followed by Tukey’s post-hoc comparison. At least three independent replicates were done.

We here collected information from an individual diagnosed with ASD at the age of seven. This patient displayed a low willingness to interact with peers, limited interests and a range of stereotypical behaviors such as mumbling and spinning toys. He also manifested mild intellectual disability. Using our published panel [14] for WGS, we identified a de novo heterogenous mutation c.87C>G on SHANK2 (NM_133266.5), a major risky gene for ASD [2, 17–23] (Fig. S1), which was verified by Sanger sequencing (Fig. 1a). Upon detailed bioinformatics annotation of the WGS data, we confirmed that this variant is a candidate mutation. This C to G single nucleotide mutation on one allele located at exon 1 of SHANK2B transcript variant, resulted in a premature stop codon, rendering SHANK2B p.Y29X (SHANK2B^*Y29X*^) (Fig. S2).

The patients’ profound neurodevelopmental deficits are reminiscent of the known role of SHANK2 gene. We hence sought to delineate the potential neurodevelopmental effects of this novel mutation. To explore the ASD-associated cellular phenotypes resulting from SHANK2B^*Y29X*^ mutation, we first reprogrammed PBMCs from the proband and his unaffected father into iPSCs [24–26]. These were referred to as patient clone (PC) and health clone (HC), respectively. We also introduced precise G-to-C mutation on one line from PC (PC1) using the CRISPR/Cas9 technique to generate rescue clones (PC1-res) (Fig. 1a). To elucidate the impact of SHANK2B^*Y29X*^ mutation on neuronal development, we utilized iPSCs-iNeuron model and applied a modified dual-SMADi monolayer differentiation protocol [27, 28] (Fig. 1b), which enable the investigation of various neuron subtypes (Fig. S3) during early stages of neuronal development. Interestingly, we observed a significant reduction in the rate of neuronal differentiation in PC lines, compared with HC lines (Fig. S4a, b). Meanwhile, there was a notable decrease in the TH positive/MAP2 positive DA population, and these phenotypes were rescued in PC1-res lines (Fig. 1c, d). Furthermore, when we examined the dendritic arbors of subtype neurons from both groups, we discovered that the dendrite length and arborization of DA neurons were significantly decreased in PC lines comparing to HC lines (Fig. 1e, f, Fig. S4c). However, the dendritic morphology of glutamatergic neurons showed only a moderate decrease (Fig. S5a–c), while that of GABAergic neurons remained unchanged (Fig. S5d–f). Electrophysiological analysis uncovered decreased membrane resistance, increased cell capacitance, elevated peak sodium (I_Na_) threshold, and reduced I_Na_ current in PC1-derived DA neurons. These abnormalities were rescued in PC1-res1 cells (Fig. 1g, h). These results demonstrate that SHANK2B^*Y29X*^ mutation affects both DA differentiation, dendritic morphology and neuronal activity.

**Fig. 2:**
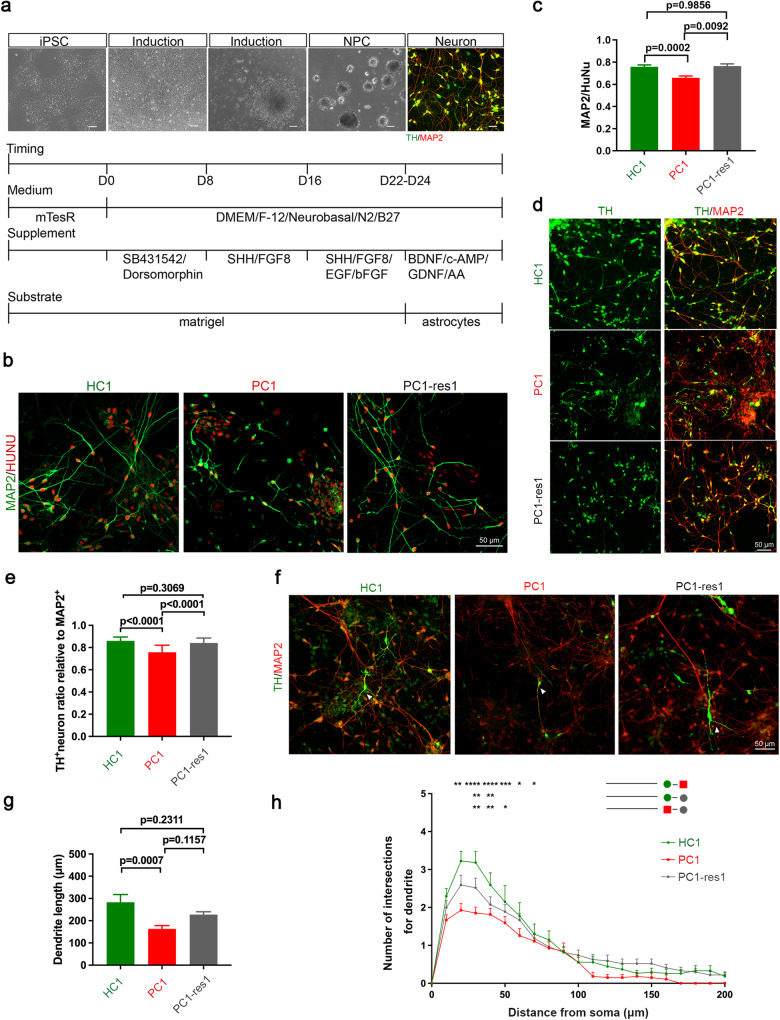
The SHANK2B^*Y29X*^ mutation from the ASD proband affects the early development of DA neurons in DA-directed differentiation protocol. **a** Schematic representation of the SHH protocol to generate a high proportion of DA neuron subtype, as assessed by ICC staining of the neuronal marker MAP2 (red) and DA neuron marker TH (green). Scale bar = 50 μm. **b** Immunostaining of HUNU (red) and MAP2 (green) in neurons harvested 9 days differentiation from NPCs. Scale bar = 50 μm. **c** Neuronal differentiation potential, determined by the proportion of MAP2 positive cells normalized to HUNU positive cells, was decreased in PC1 compared with HC1 clone, which could be rescued by genetic correction, as observed in PC1-res1 cells. **d** Immunostaining of TH (green) and MAP2 (red) in neurons harvested 9 days differentiation from NPCs. Scale bar = 50 μm. **e** Differentiation potential of DA neuron was decreased in PC1 clone compared with HC1 clone. This differentiation defect could be rescued by genetic correction, as observed in PC1-res1 clone (*n* ≥ 10 image fields from each clone were analyzed). **f** Immunostaining of TH (green) and MAP2 (red) in neurons harvested 9 days differentiation from NPCs. Scale bar = 50 μm. Analysis of dendritic length (**g**) and arborization (**h**) of TH-positive DA neurons. Neuron reconstruction based on MAP2 signal was performed using neurolucida 360 and analyzed by Sholl analysis (*n* = 30 cells in HC1, 36 in PC1 and 27 in PC1-res1clone). Data are represented as the mean ± SEM. Statistical significance was evaluated by One-way ANOVA (**c**, **e**, **g**) followed by Tukey’s post-hoc comparison and Two-way ANOVA (**h**) followed by Šídák’s multiple comparison. *p < 0.05, **p < 0.01, ***p < 0.001, ****p < 0.0001. At least two independent replicates were done.

Then we followed a SHH protocol [27, 29, 30] (Fig. 2a) to generate more DA neurons and decipher whether the DA neuron phenotypes in modified dual-SMADi monolayer differentiation protocol could be reproduced. We found that MAP2 positive/HuNu positive neuron (Fig. 2b, c), TH positive/MAP2 positive DA population (Fig. 2d, e), the dendrite length and complexity of DA neurons (Fig. 2f–h) were significantly reduced in PC lines, compared with HC neurons, and these phenotypes could be rescued in PC1-res lines.

### SHANK2B^*Y29X*^ KI phenocopies the DA neuron developmental obstacle in SHANK2B^*Y29X*^ carrier

**Fig. 3:**
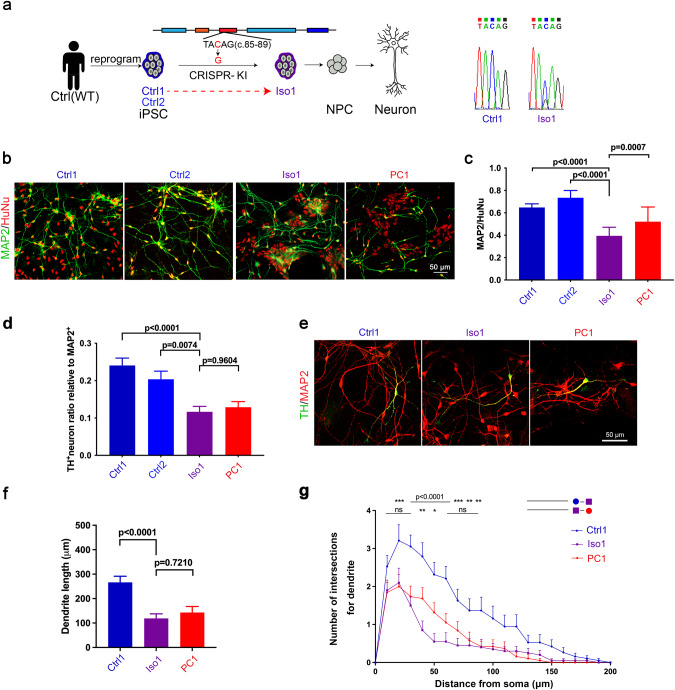
SHANK2B^*Y29X*^ KI in iPSCs-iNeuron model exhibits developmental defects of DA neurons. **a** Two iPSCs cell clones (Ctrl1, Ctrl2) were derived from a healthy individual (male) independent of this ASD pedigree, and SHANK2B^*Y29X*^ mutation was introduced into the Ctrl1 clone by the CRISPR/Cas9 technique, giving the Iso1 cell line. The heterozygous C87G mutation in the Iso1 clone was confirmed by Sanger sequencing. **b** Immunostaining of HUNU (red) and MAP2 (green) in neurons harvested 9 days differentiation from NPCs. Scale bar = 50 μm. **c**, The Iso1 clone showed a decreased potential for neuronal differentiation compared to Ctrl clones, similar to the PC1 clone (*n* ≥ 10 image fields from each clone were analyzed). **d** The differentiation potential of dopamine neurons was decreased in the Iso1 clone compared to Ctrl clones, similar to the PC1 clone. (*n* ≥ 10 image fields from each clone were analyzed). **e** Immunostaining of TH (green) and MAP2 (red) in neurons harvested 9 days differentiation from NPCs from Ctrl1, Iso1 and PC1 clones. Scale bar=50 μm. Analysis of dendritic length (**f**) and arborization (**g**) of TH-positive DA neurons. Neuron reconstruction based on MAP2 signal was performed using neurolucida 360 and analyzed by Sholl analysis. (*n* = 19 cells in Ctrl1, 25 in Iso1, and 19 in PC1 clone). Data are presented as the mean ± SEM. Statistical significance was evaluated by One-way ANOVA (**c**, **d**, **f**) followed by Tukey’s post-hoc comparison and Two-way ANOVA (**g**) followed by Šídák’s multiple comparison, respectively. *p < 0.05, **p < 0.01, ***p < 0.001, ****p < 0.0001.

To further confirm the genotype-phenotype link, we constructed isogenic c.87C>G heterozygous mutant iPSCs (Iso) from control iPSCs (Ctrl) derived from a healthy donor (Fig. 3a). Neuronal differentiation (Fig. 3b, c), predominantly the TH positive DA neuron, was attenuated in Iso line comparing to Ctrl lines (Fig. S6, Fig. 3d–e). The dendrite length and complexity of Iso1-derived DA neurons also recapitulated those of PC1 derived neurons (Fig. 3f–h). Genetic correction of the mutants rescued, while isogenic cell line knocked in with SHANK2B^*Y29X*^ mutant phenocopied those DA defeats, which validate that *SHANK2* c.87C>G mutation is primarily responsible for the DA neuron-related phenotypes in SHANK2B^*Y29X*^ carrier.

### SHANK2B^*Y29X*^ preferentially affects ALDH1A1 negative DA neurons

**Fig. 4:**
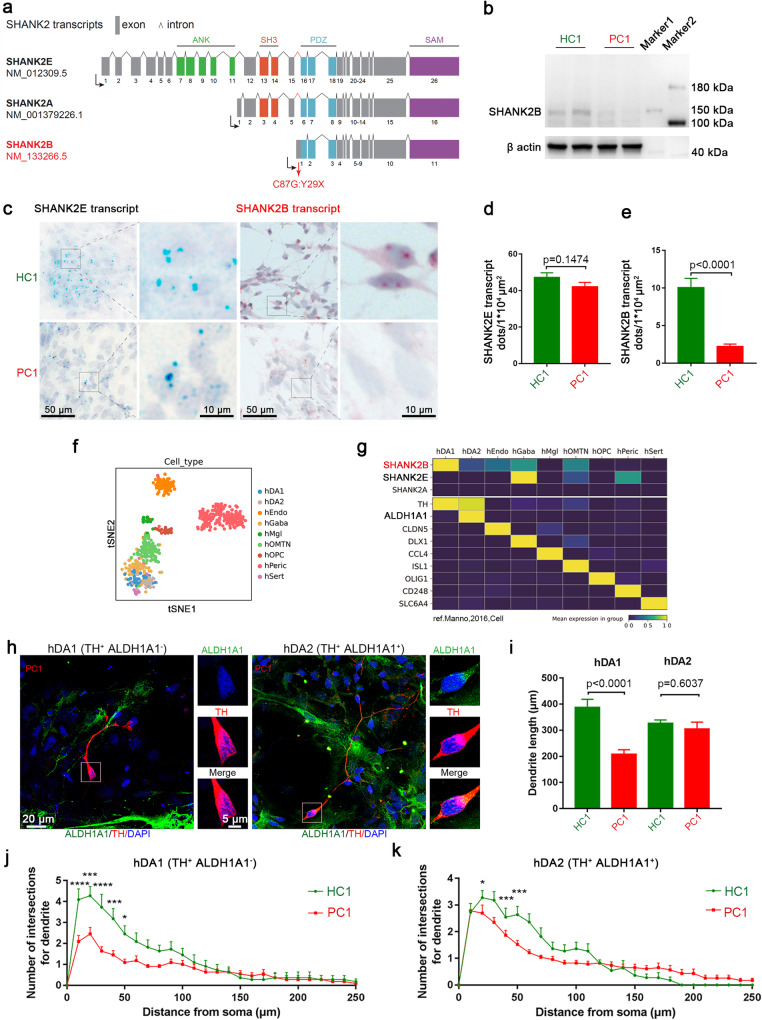
SHANK2B transcript-enriched ALDH1A1 negative hDA1 neurons were affected by SHANK2B^*Y29X*^ mutation. **a** Diagram illustrating human SHANK2 protein isoforms, SHANK2E, SHANK2A and SHANK2B. Three different promoters (black arrow) are recruited for the expression of SHANK2E, SHANK2A and SHANK2B. SHANK2E represents the longest isoform of SHANK2, containing ANK, SH3 PDZ and SAM domains. SHANK2A contains SH3, PDZ and SAM domains while SHANK2B contains PDZ and SAM domains (SHANK2A and SHANK2B were defined based on Jiang and Ehlers [4] and Eltokhi et al. [49]). The SHANK2B^*Y29X*^ mutation (red arrow) is located in SHANK2B exon area, while in intron areas of SHANK2E and SHANK2A. **b** Western blot of SHANK2 (antibody from Thermofisher PA5-39454) in neurons 5 days after differentiation from NPCs derived from HC1 and PC1 clones. β actin was chosen as reference protein. **c** Representative BaseScope images showing expression of SHANK2E transcript (blue dot) and SHANK2B transcript (red dot) in neurons 5 days after differentiation from NPCs derived from HC1 and PC1 clone. Scale bar as indicated in figures. **d**, **e**, Analysis of the number of transcript dots per 1 × 10^4^ µm^2^ area from each clone. SHANK2E signal remained unaffected (**e**) while SHANK2B signal is almost diminished (**f**) in PC1 clone. (areas included in analysis: (*n* = 28 in HC1, and 26 in PC1 SHANK2E transcript; (*n* = 35 in HC1 and 26 in PC1 for SHANK2B transcript). **f** Visualization of 9 clusters (terminally differentiated cells selected) resolved by unsupervised clustering analysis with t-SNE from human ventral midbrain single-cell transcriptomes. **g** Single-cell RNA sequencing shows high expression of SHANK2B transcript in ALDH1A1 negative hDA1 neurons, but much lower expression in ALDH1A1 positive hDA2 neurons. **h** Immunostaining of ALDH1A1 (green) and TH (red) to represent ALDH1A1 negative hDA1 neuron and ALDH1A1 positive hDA2 neuron from PC1 clone. Scale bar as indicated in figures. **i–k** SHANK2B^*Y29X*^ mutation preferentially affected dendritic morphology of hDA1 neurons. Dendritic length (**j**) and arborization (**k**) of hDA1 neurons are dramatically decreased in PC1 compared with HC1 ones, while those of hDA2 are moderately affected in PC1. (*n* = 10 hDA1, 10 hDA2 in HC1; 27 hDA1, 23 hDA2 in PC1 clone). Data are presented as the mean ± SEM. Statistical significance was evaluated by unpaired Student’s t-test (**b**, **c**, **e**, **f**, **j**) and Two-way ANOVA (k,l) followed by Šídák’s multiple comparison, respectively. *p < 0.05, **p < 0.01, ***p < 0.001, ****p < 0.0001.

SHANK2 gene can produce multiple transcripts and different protein isoforms may have specific functions. The *SHANK2* c.87C>G mutation derived from the pedigree in this study located on exon of SHANK2B (NM_133266.5) but intron of SHANK2A and SHANK2E (Fig. 4a). SHANK2B retains the PDZ domain, which is crucial for neurodevelopment [5, 7, 8, 31, 32]. Utilizing this valuable case, we first verified SHANK2B deficiency in PC1-derived neurons by western blot (Fig. 4b), whereas SHANK2E and SHANK2A isoforms were weakly detected. Therefore, isoform-sensitive BaseScope probes were used to further demonstrate the alternation of different isoforms at RNA level. Results showed reduced SHANK2B transcript but unchanged SHANK2E transcript in PC1-derived neurons (Fig. 4c–e), while this reduction could be rescued in PC1-res1 line (Fig. S7a–c). Moreover, co-staining with TH enabled the visualization of decreased SHANK2B expression in patient-derived DA neurons (Fig. S7d, e). Besides, SHANK2A signal was faintly detected in neurons 5 days differentiated from NPCs (Fig. S7f), but more pronounced at iPSCs stage when we found no significant changes between PC1 and HC1 clone (Fig. S7g, h). Bulk RNA sequencing revealed decreased SHANK2 and SHANK2B transcript expression during early neuronal stage (Fig. S8a). In a parallel manner, qRT-PCR using two different primers indicated SHANK2A and SHANK2E transcript were increased significantly (Fig. S8b), while SHANK2B was decreased (Fig. S8c). These evidence demonstrated significant SHANK2B reduction at RNA and protein levels in neurons induced from ASD patient clone (PC1) when compared with unaffected father clone (HC1) during early neural developmental stage, whereas SHANK2E and SHANK2A remained unchanged or possibly compensatory increased at different developmental stage.

To dissect the SHANK2B-specific function, we reanalyzed single-cell RNA-seq data of human ventral midbrain [16] obtained by SMART-Seq2, permitting isoform-specific transcriptome analysis (Figs. 4f, g and S9). Of note, we found that SHANK2B is mainly expressed in the ALDH1A1 negative TH positive DA neuron subtype, hDA1 [16]. In contrast, SHANK2E is expressed more in the hGABA neurons while SHANK2A expression is relatively low at the time point in midbrain examined. The *SHANK2* c.87C>G mutation dramatically affected the dendrite length and complexity of hDA1, identified by ALDH1A1 and TH co-staining, Instead, only subtle changes in dendritic arborization were observed in the ALDH1A1 positive TH positive hDA2 neurons (Fig. 4h–k).

### SHANK2B^*Y29X*^ KI mice show abnormal social behavior and DA neuron defects in VTA

**Fig. 5:**
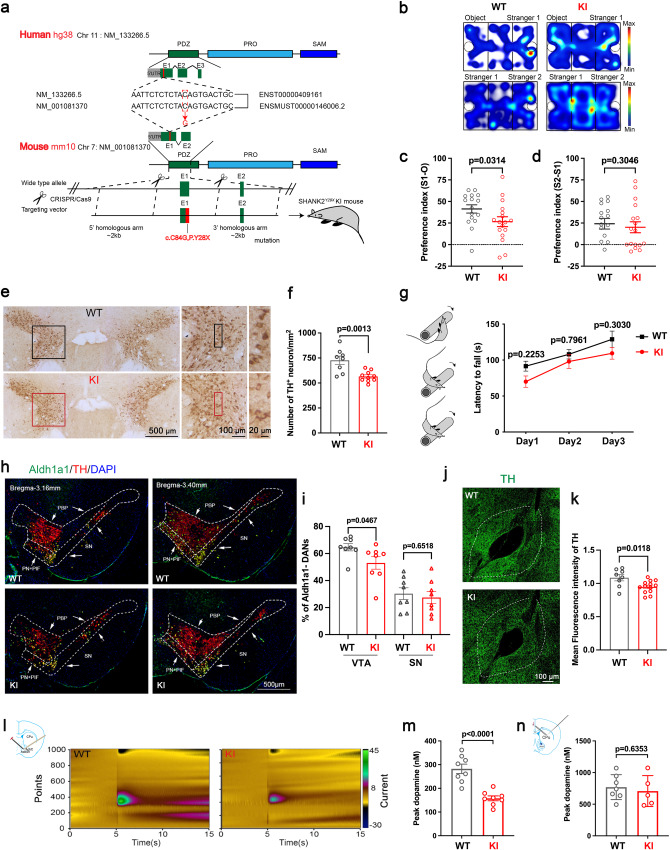
SHANK2B^*Y29X*^ KI mouse model shows deficits in social behavior and Aldh1a1 negative DA neurons in VTA. **a** Generation strategy for SHANK2B^*Y29X*^ KI mice (KI). The human *Y29X* mutation was parallel to mouse *Y28X* mutantion (NM_001081370) located on chromosome 7, which was knocked in to wild-type (WT) mice, resulting in the generation of KI mice. **b** Representative heat map displaying traces from WT and SHANK2B^*Y29X*^ KI mice in the three-chamber social assay. Preference ratio in the second phase (**c**), and third phase (**d**) in the three-chamber social assay, representing impaired social preference and intact social recognition memory, respectively, in homozygous SHANK2B^*Y29X*^ KI mice. (*n* = 16 WT mice and 17 KI mice, 5-week-old). **e** DAB staining of TH in coronal midbrain sections of WT and KI mice. Scale bar as indicated in figures. **f** Quantitative analysis of number of TH+ neurons in VTA per mm^2^ of WT and KI mice (*n* = 8 brain slices from 3 WT mice and (*n* = 10 from 3 KI mice, 8–9 week–old). **g** SHANK2B^*Y29X*^ KI mouse exhibited normal motor learning ability in the rotarod test (*n* = 10 WT mice and (*n* = 11 KI mice, 8-week-old). **h** Immunostaining of Aldh1a1 (green) and TH (red) in VTA and SN of WT and SHANK2B^*Y29X*^ KI mice. Dashed lines and arrows indicate SN and VTA (including PBP, PIF, PN) from brain sections (bregma: −3.16 mm and −3.40 mm). Scale bar=500 μm. **i** Quantitative analysis of percentage of Aldh1a1 negative dopamine neuron in VTA and SN (*n* = 8 brain slices from 3 WT mice and 3 KI mice, 8-week-old). **j** Immunostaining of TH in nucleus accumbens (NAc) of WT and SHANK2B^*Y29X*^ KI mice. Scale bar=100 μm. **k** Mean fluorescence intensity of TH in NAc of WT and SHANK2B^*Y29X*^ KI mice (*n* ≥ 10 image fields from 3 WT mice and 3 KI mice, 8-week-old). **l**, **m** Representative traces of FSCV records and quantitative analysis of dopamine transmitter release in the NAc evoked by electrical stimulation in VTA area. The recorded current peaks (detected by microelectrode in NAc) are analyzed to determine the concentration of dopamine release by comparing the current measurements to a calibration curve (*n* = 8 brain slices from 3 WT mice and 3 SHANK2B^*Y29X*^ mice). **n** A schematic of FSCV records and quantitative analysis of dopamine transmitter release in the dorsal striatum (CPu) evoked by electrical stimulation in SN area (*n* = 7 brain slices from 3 WT mice and 5 brain slices from 3 KI mice). Data are presented as the mean ± SEM, Statistical significance was evaluated by Mann–Whitney test (**c**). Two-way ANOVA (**g**) followed by Šídák’s multiple comparison and two-tailed unpaired Student’s *t*-test (**d**, **f**, **i**, **k**, **m**, **n**), correspondingly.

To further investigate how SHANK2B^*Y29X*^ impacts in vivo brain development at the cellular and behavioral levels, we generated a SHANK2B^*Y29X*^ KI mouse (Fig. 5a). The targeting vector containing mouse p.Y28X (corresponding to human Y29X, as described in Method) was knocked into the endogenous *Shank2* locus through CRISPR/Cas9 technology and homologous recombination. Heterozygous male and female KI mice were mated to produce the homozygote mutants. The offspring was born to the Mendelian ratio but decreased body weight was apparent in KI mice (Fig. S10a). We next explored whether the homozygous KI mice were conferred with autism-like phenotype. In the three-chamber social assay (Fig. 5b–d), WT mice showed preference towards a stranger over an object, whereas KI mice showed no observable inclination in second phase, indicating the KI mice exhibited deficits in sociability, which was parellelly probed in homecage social test (Fig. S10b, c). We also noticed that KI mice showed abnormal stereotypic behaviors, hyperactivity, anxiety and impaired spatial learning and memory ability (Fig. S10d–j). In sum, we demonstrated that the *SHANK2* c.87C>G mutation is a pathogenic mutation, which is sufficient and essential to induce autistic-like behaviors in mice. We then focused on VTA, which contains abundant DA neurons that form the mesolimbic dopamine system [33]. Echoing the phenotypes of the in vitro model, reduced number (Fig. 5e, f) and shortened dendrite length (Fig. S10k–m) of TH+ neurons in the VTA were observed. Besides, synaptic dysregulation, including decreased dendritic spine density (Fig. S10n–o), concomitant with increased mEPSC amplitude and frequency (Fig. S10p–q) were detected in VTA neurons of KI mice.

Regarding the distinct impacts of SHANK2B deficiency on ALDH1A1 positive and negative DA neurons in vitro, we first demonstrated the SHANK2B protein isoform deletion and decreasd TH expression brought by SHANK2B^*Y29X*^ mutation in VTA of mice by western blot (Fig. S11a). In the anatomical level, two main populations of DA in the midbrain are located in the substantia nigra pars compacta (SN) and VTA, respectively [34, 35]. DA neurodegeneration upon Parkinson disease occurs mainly in the SN where most Aldh1a1 positive DA neurons reside [36–39]. In line with this notion, KI mice showed normal motor learning ability (Fig. 5g), the occupancy of Aldh1a1 negative DA neurons was affected in VTA rather than SN (Fig. 5h, I, Fig. S11b, c). Given that VTA projecting to NAc is, a well-known brain circuit that encode and predict key features of social behaviors [33], we wondered the projection of TH+ neurons to NAc could be altered. As expected, mean fluorescence intensity of TH in NAc (Fig. 5j, k) as well as electric stimulation-induced NAc release probed by FSCV (Fig. 5l–m, Fig. S11d, e) was greatly diminished in KI mice. Instead, neither TH intensity in medial prefrontal cortex and basolateral amygdala (Fig. S11f, g), nor DA release from SN to dorsal straitum was decreased significantly (Fig. 5n). Therefore, the expression of endogenous SHANK2B has fundamental function in not only dopamine neuron development in VTA but the establishment of dopamine based brain circuits.

It is worth noting that we observed both Shank2A and Shank2B isoforms were affected whereas Shank2E was relatively unchanged in cortex of homozygous KI mice by western blot (Fig. S12a). Besides, an uncharacterized novel short isoform appeared in KI mice. To further delineate the distinct effects of SHANK2B^*Y29X*^ mutation on different isoforms in vivo, we designed primers targeting Shank2A, Shank2B and Shank2E transcripts (Table S2) and performed qRT-PCR on three brain regions (Fig. S12b). Results revealed a significant decrease of Shank2A transcript across brain regions, while Shank2E only showed significant decrease in cerebellum. Shank2B remained unchanged in cortex and hippocampus, but exhibited a significant increase in cerebellum.

We further investiagetd whether the mutation also affects SHANK2 splicing in human and mouse. Point mutations can alter branch site selectivity in pre-mRNA splicing or activate a cryptic splice site in part of the transcript that usually is not spliced. Since the C > G mutation sits right adjacent to a canonical splice acceptor site (AG), it has the potential to create a new 3′ AG splice acceptor site (ACAG → AGAG). Available splice site predictor, MaxEntScan [40] (Fig. S13) and Neural network [41] (Fig. S14) both predicted the new AG splice site (2 bp away from the original splice site) in human and mouse. Sequencing data from human cell line (Fig. S15) and mouse tissue (Fig. S16) revealed that the length of splicing transcripts were differed, where 2 bp longer undefined transcript variant was found in KI mice compared with WT mice, but not found in patient clone, when we aligned our RNA-Seq reads to genome and visualized by IGV. These evidence suggested that the 2 bp shift in splicing introduces a new transcript with a premature termination codon within the affected exon, resulting in nonsense-mediated decay, which impacts Shank2A in vivo in our case. Utilizing SnapGene v4.1.8 to evaluate the potential implications of new transcript on translation, we identified an early stop codon possibly mediating the decay of Shank2A transcript (Fig. S17a). On the other hand, C > G mutation might seed the formation of novel isoform observed in Western blot by creating new open reading frame. SnapGene implied that a shorter protein isoform would be derived from 2 bp longer Shank2A transcript (Fig. S17a), as well as from mutant Shank2B transcript (Fig. S17b). These translation products might correspond to the novel short isoform. Taken together, C > G mutation not only generates an early stop codon in SHANK2B transcript, but creates a new splice acceptor site, which is predicted to affect the splicing events, resulting in shorter protein product observed in Western blot.

## Discussion

Previous reported intragenic mutations of SHANK2 derived from ASD patients affected multiple isoforms [4, 5], making it difficult and ambiguous to clarify the pathological mechanisms in an isoform-specific manner. Here, we collected a proband with a de novo single nucleotide mutation (SHANK2B^*Y29X*^) from an ASD pedigree which preferentially affected the SHANK2B isoform, while leaving other SHANK2 transcripts (SHANK2E and SHANK2A) unaffected in human cell model. We then demonstrated a significant early neuronal developmental obstacle in patient-derived clones, in consistent with previous studies on Shankopathy [11, 15]. More importantly, dominant detrimental effects were posed on DA neurons among which differentiation ratio, dendritic length and complexity, and electrophysiological properties were all adversely affected. Integrating the transcriptome database of the human midbrain [16], we unraveled the abundance of SHANK2B in the ALDH1A1 negative DA neurons while SHANK2B^*Y29X*^ mutation impaired the development of this DA subpopulation in vitro and in vivo. Taken together, our study sheds new light on the critical role of SHANK2B isoform in early dopamine neuron growth and functioning.

Global transcriptional analysis has revealed the differential distribution of isoforms, especially synapse proteins, among different neuronal subtypes, across brain regions and developmental stages [42, 43]. SHANK genes can undergo alternative splicing and utilize different promoters to generate multiple mRNA transcripts and corresponding protein isoforms, some of which lack certain domains, giving functional diversity [4, 7, 44, 45]. Indeed, there have been some studies on Shank3 isoforms supported this concept [44, 46, 47]. Regarding the expression of SHANK2 isoforms, SHANK2E, the longest isoform, is mainly expressed in cereblleum in both mouse and human brain. On the other hand, SHANK2B and SHANK2A are ubiquitously expressed in various areas except cerebellum, such as cortex, frontal lobe and hippocampus in human. In these areas, SHANK2B is expressed more prominently than SHANK2A. Besides, our BaseScope results indicated low expression of SHANK2A during early neuronal developmental stage (from NPCs to immature neurons), whereas SHANK2E and SHANK2B are more prominent. However, functional study on specific isoforms are limited. A recent study demonstrated the role of SHANK2A in redistribution of Ca^2+^-permeable AMPA receptors between apical and basal hippocampal CA1 dendrites, leading to impaired synaptic plasticity in the basal dendrites. SHANK2A overexpression resulted in impaired social interaction and odor processing [48]. Therefore, mutation that aberrantly disrupts transcription of SHANK or following translation of isoforms might lead to varied molecular phenotypes and eventually divergent behavioral outcomes observed in animal models and clinical patients [5, 49].

Concerning the pronounced difference in altered isoform expression between in vitro and in vivo settings, wherein SHANK2B isoform was affected in human cell line but Shank2A and Shank2B were disrupted in mouse brain, we here provided possible explanation. Aberrant alternative splicing events due to shifting splicing site upon point mutation are predictable in both species. However, such perturbation impacted more extensively in homozygous mutant mice where Shank2A transcription in addition to Shank2B translation were adversely affected, compared with heterozygous mutant neurons. Notably, a novel isoform, detected by C-terminus recognizing antibody in KI mice, with smaller molecular size than Shank2B, shall be lack of PDZ domain that is essential for Shank2 function (Fig. S17). Moreover, it might interact with different Shank2 isoforms or other Shank proteins (Shank1, Shank3) within the synapse or cellular compartments. Eltokhi et al. have pointed out the possibility of residual expression of truncated proteins or mRNAs, which could potentially disrupt the structure or flexibility of the SHANK scaffold [49, 50]. Whether undefined transcript or isoform exert compensatory function or adverse effects require evidence.

Since the disruption of various isoforms in animal models, we also explored whether the SHANK2B^*Y29X*^ mutation affect other neuron subtypes, such as excitatory (glutamatergic) and inhibitory (GABA) neurons in cortex and hippocampus. Both qRT-PCR of Gad1, Gad2 (Fig. S18a–d), which encode the proteins involved in GABA synthesis; and IF staining (Fig. S18e, f) of VGAT, which is critical for vesicular GABA transport, hinted dysregulation of inhibitory neurotransmission of GABAergic neurons in cortex. However, no significant changes were observed in cortex in vGlut1 protein expression upon SHANK2B^*Y29X*^ mutation (Fig. S18g–k). The difference in affected isoforms and neuron subtypes might partially contribute to the behavioral differences observed in KI mouse and autistic patient in our case, since hyperactivity, aggressive behavior (data not shown) were unexpectedly observed in mouse model. Therefore, convergent results render the SHANK2B^*Y29X*^ KI mouse model to be a multi-hit mouse model of ASD. The identification of mutation-targeted cell-type drives the mechanistic revelation and therapeutic treatment of the disease.

SHANK2 proteins belong to scaffolding proteins that tether and organize intermediate scaffolding proteins located at the PSD of excitatory synapses with implication in synaptic formation, development, and plasticity [5, 31]. Since PDZ and SAM domains within brain SHANK2 isoforms mediate the formation of compact postsynaptic nanocluster [51], individuals or animal models carrying SHANK2 mutations disrupting various isoforms often exhibit altered synaptic/spine number [8, 21, 52], as well as impaired synaptic plasticity [22] and transmisstion [21]. In line with these observations, our study revealed a decrease in spine, whereas increase in mEPSC frequency and amplitude in VTA of SHANK2B^*Y29X*^ KI mice. It is important to note that the VTA receives input from various regions and harbors multiple neuron identities (DA and GABA neurons) [53], which distinguishes it from glutamatergic neurons studied mostly in previous investigations. This discrepancy in brain regions or probed neuronal subtypes may contribute to the conflicting results observed. In addition, the small sample size in our study necessitates additional evidence to elucidate the impacts of SHANK2B^*Y29X*^ mutation on synaptic electrophysiology.

Besides synapse deficit, neuronal growth deficit is commonly observed in Shankopathy [8, 11]. Constitutive SHANK2 knockout [21, 22, 52, 54] and conditional SHANK2 deletion in certain cell type [55–57] brought divergent behavioral abnormalities in social, repetitive, locomotor, anxiety and hyperactivity domains in mouse models, hinting the distinct role of SHANK2 in different neuron subtypes and brain areas that contributes to diverse brain functions [49]. A recent study demonstrated the expression of SHANK2 in TH positive neurons in both human and mouse brain [58]. Combined with the SMART-seq human dataset, we could hypothesize that SHANK2B is expressed in TH positive neurons in midbrain and affect the DA neuron growth and functioning. Mesolimbic DA pathway has been extensively studied in resislience [59] and social reward [33, 60]. Deciphering the intrinsic properties and functionality of Aldh1a1 in dopamine, large amount of studies have correlated reduction of Aldh1a1 expression or substantial loss of Aldh1a1 positive DA neurons selectively in SN to etiopathogenesis of PD, indicating this distinct neuron subpopulation as a crucial role in motor skill learning [37, 61, 62]. Accordingly, neither Aldh1a1 positive DA neurons nor SN DA projection to straitum responsible for motor function were disturbed in our case, which align with the in vitro study. On the other side, VTA DA projecting to NAc was greatly attenuated. In this sense, we provided novel insights into the critical role of Shank2 in development of Aldh1a1 negative DA neurons and integrity of the DA-based VTA-NAc circuit. In another study, correcting VTA dopaminergic activity during adolescence can alleviate social deficits in Shank3-mutant mice [63]. These collaborative findings establish a strong link between dopamine and Shankopathy.

For further implementation and translation, 1. whether optogenetic/chemogenetic activation of DA neuron in VTA could improve behavioral abnormalities observed in knockin mice; 2. whether selective SHANK2B^*Y29X*^ knockin within DA subpopulation would displayed comparable phenotypes in mice; 3. whether other disease-causing mutations of SHANK2 (e.g., R462X, T1127M, and L1008_P1009dup) [2, 6–8, 48, 64] disrupting multiple isoforms of SHANK2 affect ALDH1A1 negative DA neurons, warrants further investigation.

In all, despite an overwhelming consensus on the importance of studying isoform functions of SHANK, rare studies are able to interpret the functional relevance between particular isoform and phenotypic consequence due to limited identified SHANK2 mutants targeting single isoform. Our study, for the first time, demonstrated that the isoform-specific expression of SHANK2B in the ALDH1A1 negative DA neurons, in which the loss-of-function mutation of SHANK2B derived from ASD patient impairs their growth and function in vitro. The characterization of the isoform carrying patient-derived mutation would empower the understandings of heterogeneous and complex ASD genetics [5].

## Supplementary information

Supplementary figures and tables

## Data Availability

All data generated for this manuscript have been included in this article. The bulk RNA sequencing data and single cell sequencing data have been deposited in public repository, the accession number is GSE256210. All other data are available from the corresponding author upon reasonable request.
