# Supplementary material for: Autism patient-derived SHANK2BY29X mutation affects the development of ALDH1A1 negative dopamine neuron
Source: Mol Psychiatry. 2024 May 4;29(10):3180–94. doi: 10.1038/s41380-024-02578-6 (PMC11449796; doi:10.1038/s41380-024-02578-6)
Supplement: Supplementary file 1 — Supplementary figures and tables [file 41380_2024_2578_MOESM1_ESM.pdf]

Autism patient-derived SHANK2B^{Y29X} mutation affects the development of ALDH1A1 negative dopamine neuron

Supplementary figures and tables

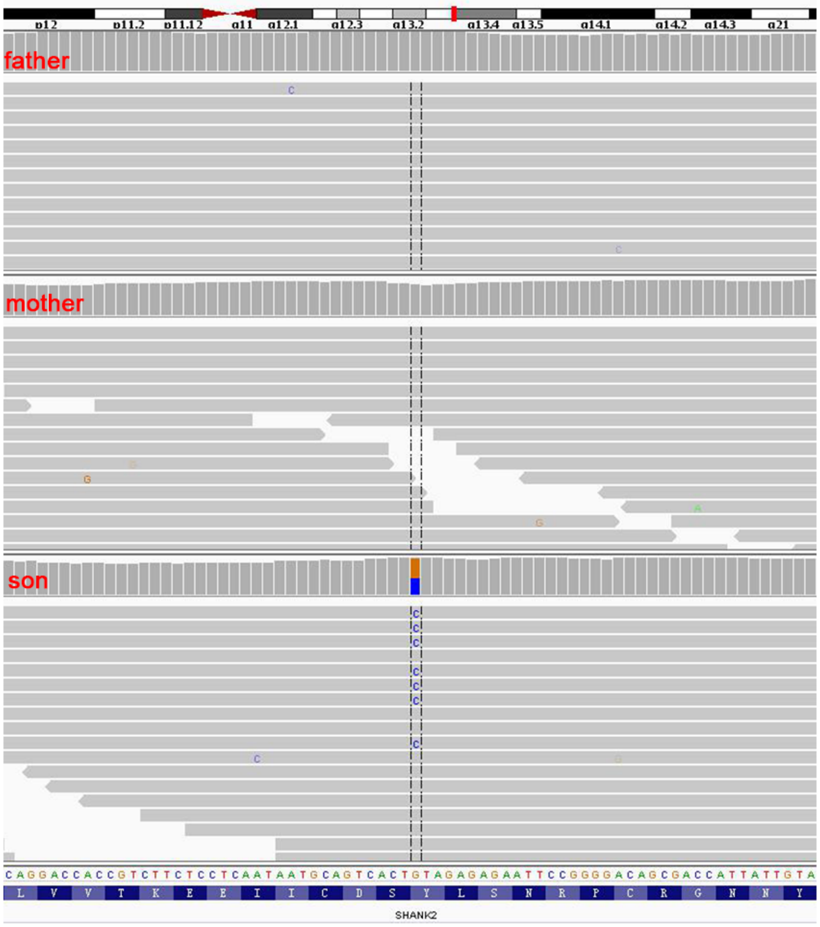

Figure S1. Identification of SHANK2B^{Y29X} mutation.

Whole-genome sequencing revealed a heterozygous stop-gain mutation (c.C87G; p.Y29X) in the SHANK2 gene (NM_133266) of the autism patient (son). This mutation was not found in his unaffected parents. Manual inspection confirmed the absence of other stop-gain mutations or frameshift indels. No other de novo single nucleotide variant (SNV) mutations associated with autism spectrum disorder was found.

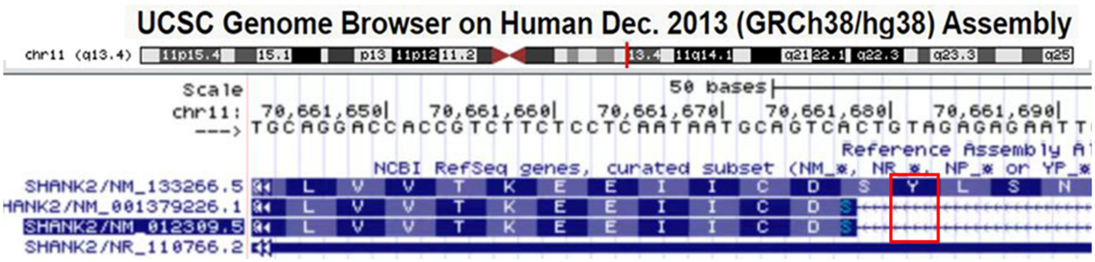

Figure S2. The location of SHANK2B^{Y29X} mutation.

The C87G:p.Y29X mutation is located in exon 1 of SHANK2B (NM_133266.5) and in the intron regions of SHANK2E (NM_012309.5) and SHANK2A (NM_001379226.1), as annotated in the UCSC genome browser.

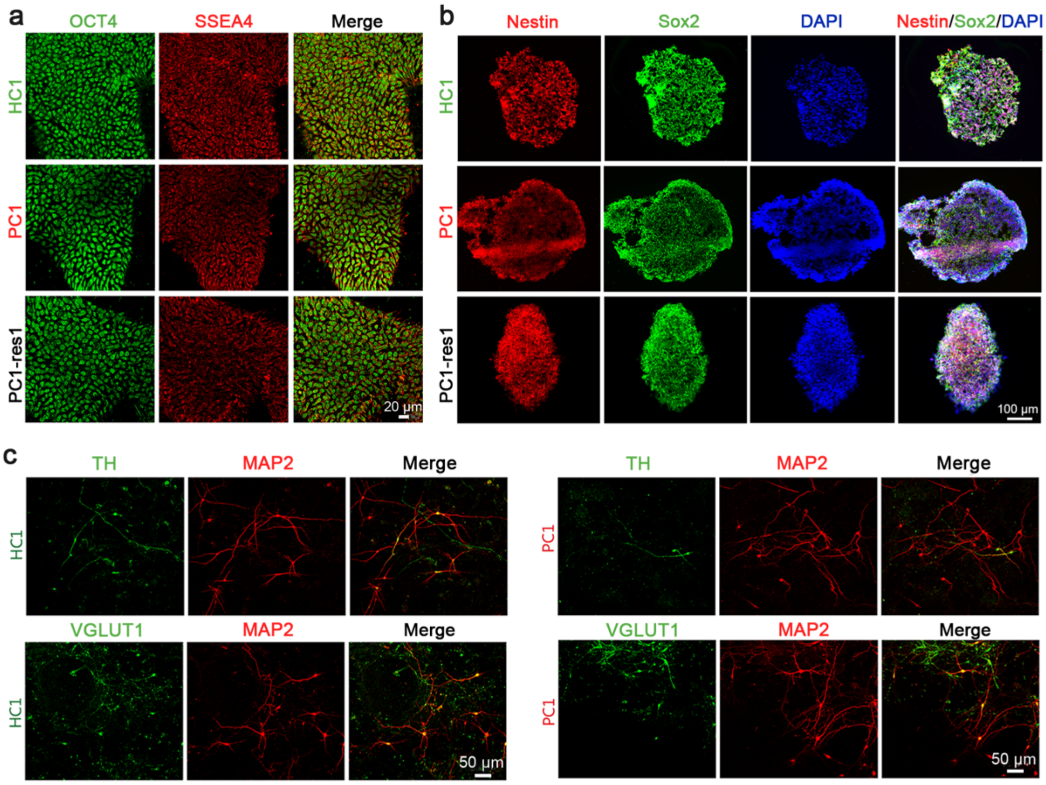

Figure S3. Characterization of iPSC-iNeuron model.

a, Immunofluorescence staining showing the expression of pluripotency markers OCT4 and SSEA4 in induced pluripotent stem cells (iPSCs) from each clone. Scale bar=20 μm.

b, Neural progenitor cells (NPCs) obtained after more than 15 days of neuronal induction from iPSCs. NPCs express SOX2 and Nestin. Scale bar=100 μm.

c, Differentiated neuron subtypes induced from modified dual-SMADi monolayer differentiation protocol, expressing neuronal marker MAP2, dopamine neuron marker TH, and glutamatergic neuron marker VGLUT1. Scale bar=50 μm.

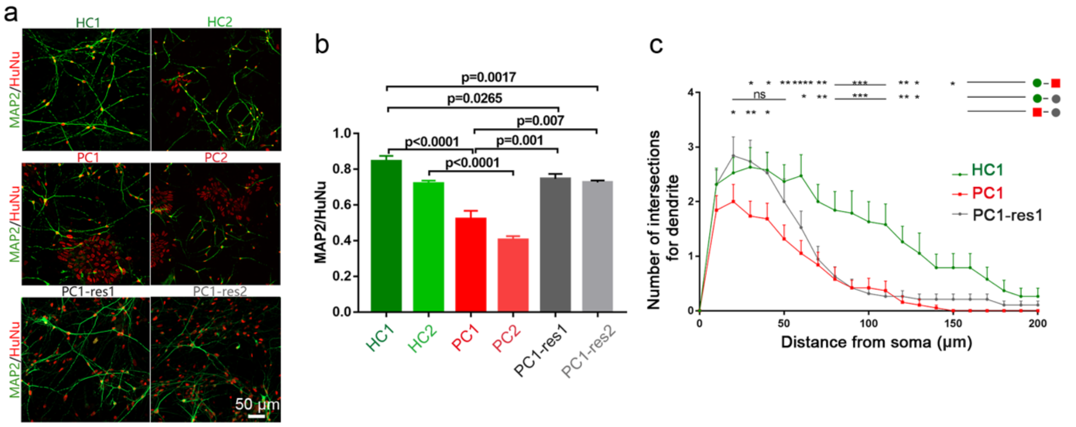

Figure S4. SHANK2B^{Y29X} carrier shows defects in neuronal differentiation potential and dendritic arborization of dopamine neuron.

a, Immunostaining staining of MAP2 (green) and HuNu (red) in iPSC-derived neurons from each clone. Scale bar=50 μm.

b, Proportion of MAP2-positive cells normalized to HuNu-positive cells, indicating neuronal differentiation potential. (n⩾8 image fields analyzed per clone).

c, Dendritic arborization in iPSC-derived TH+ neuron. (n=27 in HC1, 19 in PC1, 28 in PC1-res1 clone).

Data are presented as the mean±SEM. Statistical significance was evaluated by One-way ANOVA (b) followed by Tukey's post-hoc comparison and Two-way ANOVA (c) followed by Šídák's multiple comparison. *p<0.05, **p<0.01, ***p<0.001, ****p<0.0001, ns: not significant.

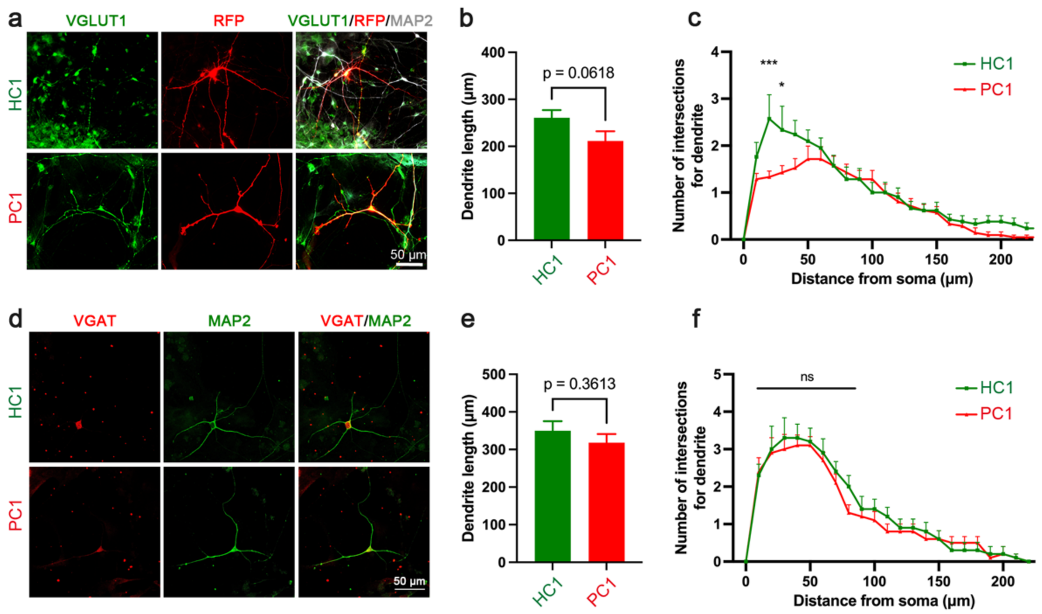

Figure S5. Dendritic morphology of glutamatergic neurons and GABAergic neurons in SHANK2B^{Y29X} carrier.

a, Immunostaining of VGLUT1 (green) and MAP2 (white) in iPSC-derived neurons from HC1 and PC1 clone. iPSC-iNeurons were transfected with control lentivirus expressing red fluorescent protein (RFP) to visualize neuronal morphology. Scale bar=50 μm.

b-c, Dendritic length analysis (b) and arborization analysis (c) in iPSC-derived VGLUT1+ neuron. Neuronal reconstructions were performed based on RFP signal using neurolucida 360, and Sholl analysis was conducted. (n=26 in HC1 and 21 in PC1 clone).

d, Immunostaining of VGAT (red) and MAP2 (green) in iPSC-derived neurons from HC1 and PC1 clone. Scale bar=50 μm.

e-f, Dendritic length analysis (e) and arborization analysis (f) in iPSC-derived VGAT+ neuron. Neuronal reconstructions were performed based on MAP2 signal using neurolucida 360, and Sholl analysis was conducted. (n=10 in HC1 and 10 in PC1 clone).

Data are presented as the mean±SEM. Statistical significance was evaluated by unpaired Student's t-test (b,e) and Two-way ANOVA (c,f) followed by Šídák's multiple comparison. *p<0.05, **p<0.01, ***p<0.001, ns: not significant.

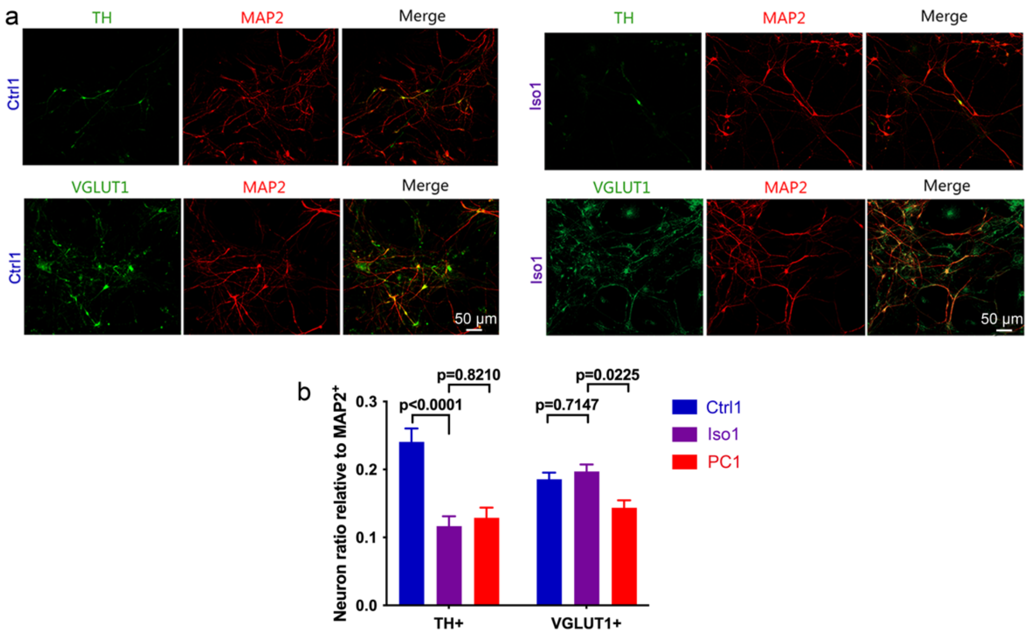

Figure S6. SHANK2B^{Y29X} mutation KI affects dopamine neuron differentiation potential.

a, Immunostaining of TH, VGLUT1 (green) and MAP2 (red) in iPSC-derived neurons from Ctrl1 and Iso1 clone. Scale bar=50 μm.

b, Differentiation potential of dopamine neuron and glutamatergic is determined by the proportion of TH positive cells and VGLUT1 positive cells normalized to MAP2 positive cells, respectively. (n⩾10 image fields from each clone were analyzed).

Data are presented as the mean±SEM. Statistical significance was evaluated by One-way ANOVA followed by Tukey's post-hoc comparison.

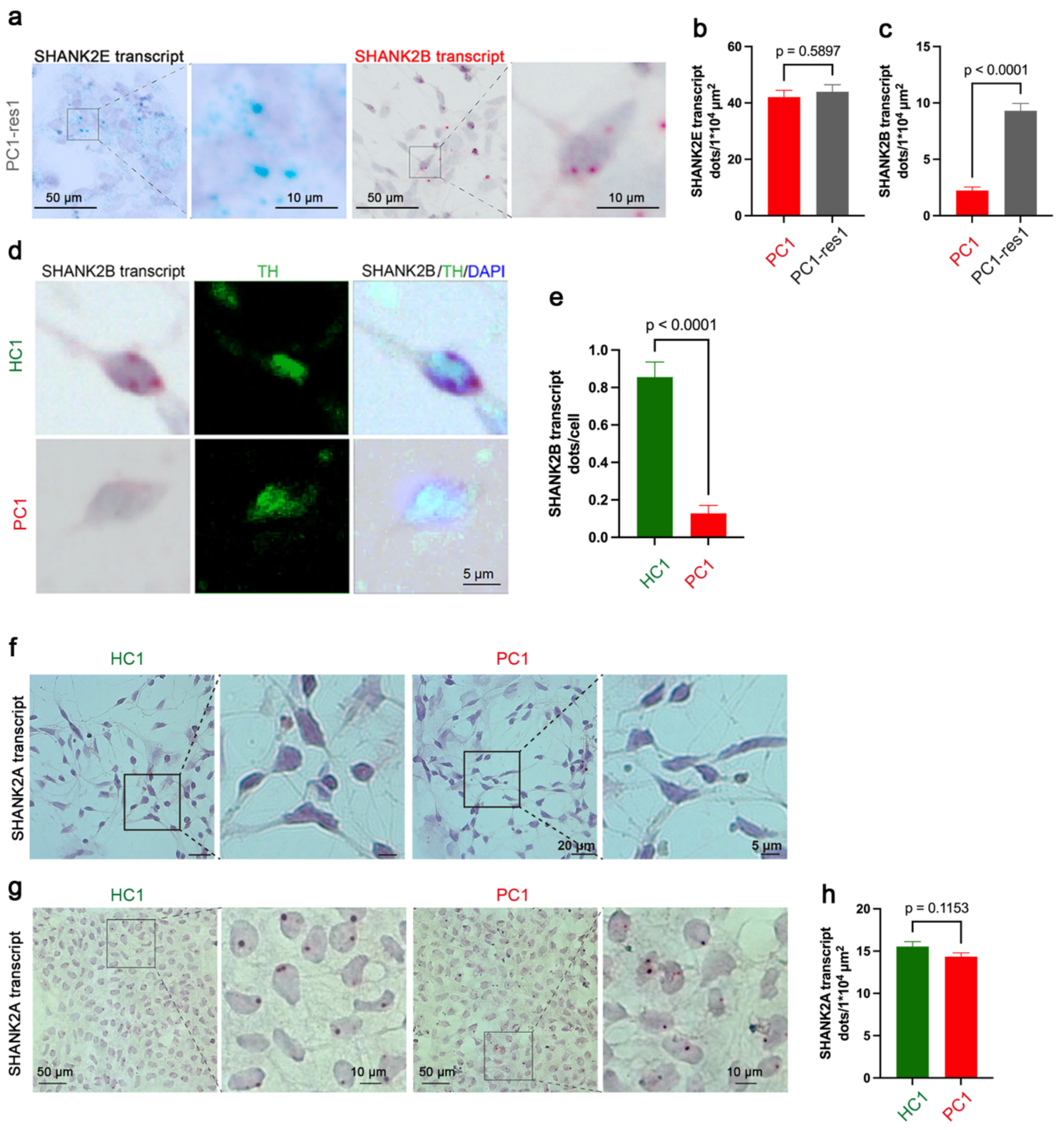

Figure S7. Reduced SHANK2B transcript expression in SHANK2B^{Y29X} carrier derived neurons.

a, Representative BaseScope images showing expression of SHANK2E transcript (blue dot) and SHANK2B transcript (red dot) in neurons harvested 5 days differentiated from NPC from PC1-res1 clone.

b, Quantitative analysis of SHANK2E transcript expression in neurons harvested 5 days differentiated from NPC from PC1 and PC1-res1 clone. n=26 image fields (1*10^4 μm^2) from PC1 and 33 from PC1-res1 were analyzed.

c, Quantitative analysis of SHANK2B transcript expression in neurons harvested 5 days differentiated from NPC from PC1 and PC1-res1 clone. n=26 image fields (1*10^4 μm^2) from PC1 and 27 from PC1-res1 were analyzed.

d, Representative images of BaseScope followed by immunohistochemistry staining probing the expression of SHANK2B transcript (red dot) in TH+ (green) cells from HC1 and PC1 clone.

Scale bar=5 μm.

e, Quantification analysis demonstrates significantly reduced SHANK2B transcript expression in TH+ cells from PC1 compared to HC1 clone. n=76 TH+ cells from HC1 and 78 from PC1 clone were analyzed.

f, Representative BaseScope images showing almost no SHANK2A transcript signals in neurons harvested 5 days differentiated from NPC from HC1 and PC1 clone.

g, Representative BaseScope images illustrating the expression of SHANK2A transcript (red dot) in iPSC from HC1 and PC1 clone.

h, Quantitative analysis of SHANK2A transcript expression in iPSC from HC1 and PC1 clone. n=30 image fields ($1*10^4$ μm^2) from each group were analyzed.

Data are presented as the mean±SEM. Statistical significance was evaluated by unpaired Student's t-test

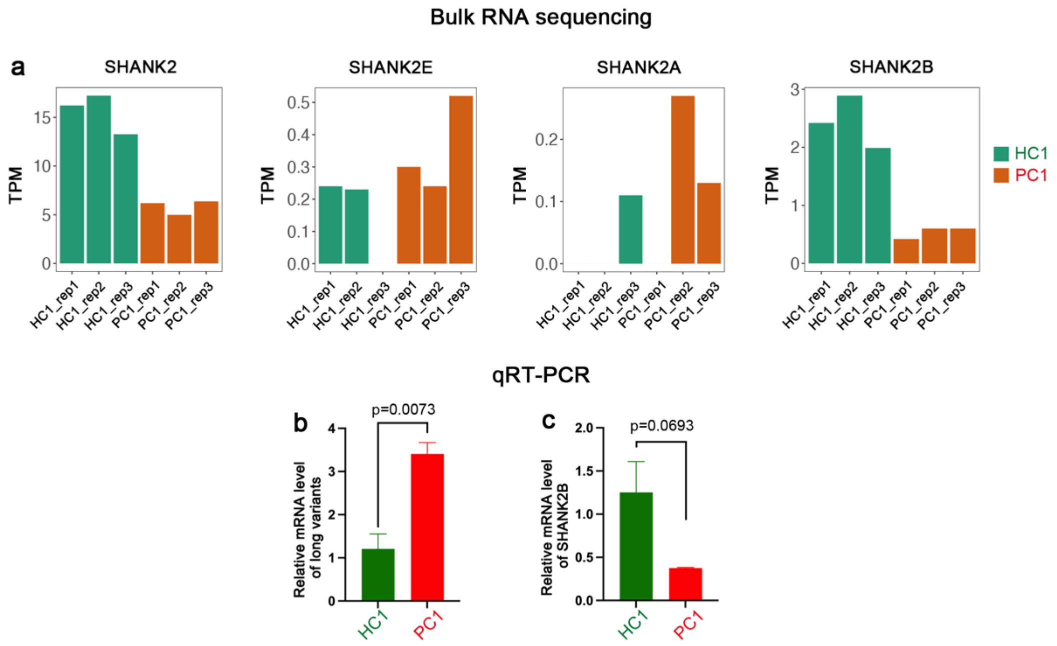

Figure S8. Altered SHANK2 transcript expression in SHANK2B^{Y29X} carrier derived neurons.

a, Bar plots showing Transcripts Per Million (TPM) values of SHANK2 gene and its isoforms SHANK2E, SHANK2A, SHANK2B transcripts obtained from bulk RNA sequencing data. Three replicates from each clone were quantified. The results demonstrate reduced expression of SHANK2 and SHANK2B transcript in neurons from autism patient clone (PC1) compared with his unaffected father (HC1). n=3 from each clone.

b-c, Relative mRNA level of SHANK2 transcript variants on neurons harvested 9 days differentiated from NPC, normalized to GAPDH. Two products (SHANK2E and SHANK2A) were generated from one primer pair (b). n=3 from each clone.

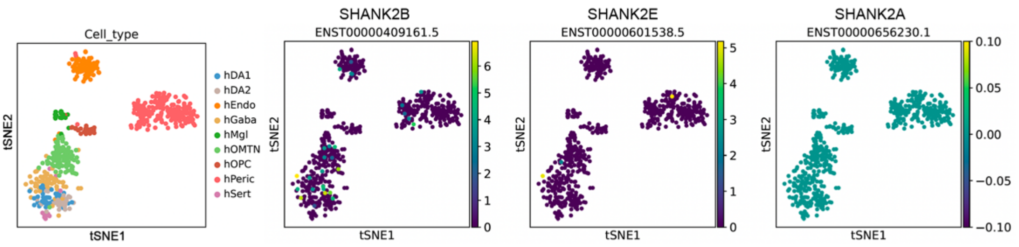

Figure S9. Expression of SHANK2 transcript variants.

Visualization of SHANK2B (ENST00000409161.5), SHANK2E (ENST00000601538.5) and SHANK2A (ENST00000656230.1) distribution with t-SNE in 9 clusters (terminally differentiated cells). SHANK2B is enriched in hDA1 cell cluster.

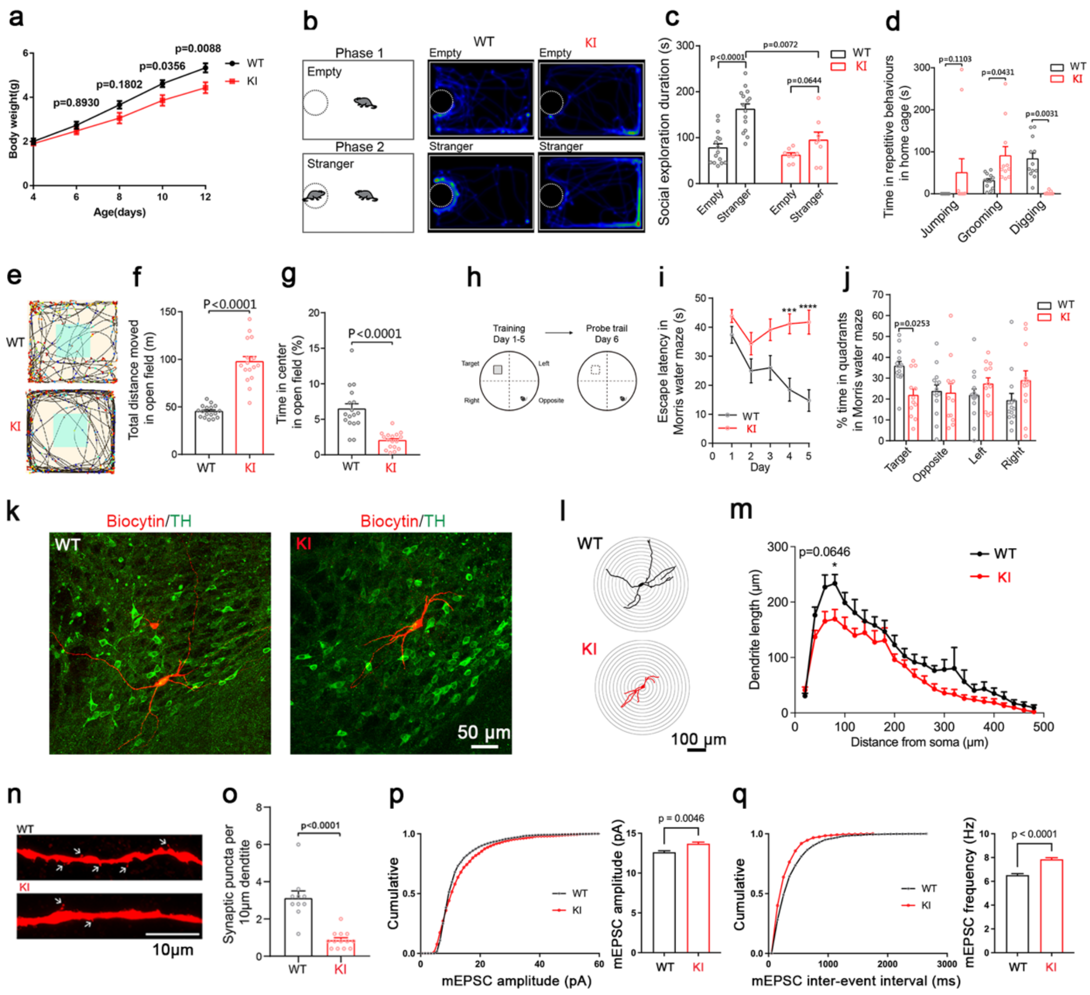

Figure S10. Behavioral and dopamine defects in homozygous SHANK2B^{Y29X} KI mice.

a, Changes in body weight during postnatal 12 days. n(WT)=5, n(KI)=5.

b, Representative traces of WT and SHANK2B^{Y29X} KI mice in each phase of the home cage social interaction test

c, Impaired social interaction of SHANK2B^{Y29X} KI mice observed in the home cage social

interaction test. n(WT)=15, n(KI)=8.

d, SHANK2B^{Y29X} KI mice display stereotyped behavior, with increased grooming behavior and decreased digging behavior in the home cage. n(WT)=12, n(KI)=11.

e, Representative traces of WT and SHANK2B^{Y29X} KI mice in the open field test.

f-g, SHANK2B^{Y29X} KI mice exhibit hyperactivity (increased total distance, f) and anxiety (decreased time spent in the center, g) in the open field test. n(WT)=14, n(KI)=10.

h, Representative traces of WT and SHANK2B^{Y29X} KI mice in the Morris water maze test.

i-j, SHANK2B^{Y29X} KI mice show poor learning performance (increased escape latency to platform) during the training trial (m) and impaired memory function (spent less time in the target quadrant) during the probe trial (n) in the Morris water maze test. n(WT)=14, n(KI)=10.

k, Representative images of TH (green) and biocytin (red) staining in ventral tegmental area (VTA) of WT and SHANK2B^{Y29X} KI mice. Scale bar as indicated in figures.

l, Reconstruction of the TH+ neuron was based on Biocytin by neurolucida 360 and analyzed by Sholl analysis.

m, Dendritic length of TH+ neuron in the VTA of WT and SHANK2B^{Y29X} KI mice. Neuron number: n(WT)=10, n(KI)=10.

n, Representative images showing dendrites of TH+ positive cells stained by biocytin (red) in the VTA from WT and SHANK2B^{Y29X} KI mice. White arrow indicates dendritic spines.

o, Decreased spine density in the VTA of SHANK2B^{Y29X} KI mice. At least three dendrite segments in each neuron were included in the analysis, and each analyzed dot represented one neuron. n⩾8 mice in each group.

p-q, Whole-cell patch clamp recording shows increased miniature excitatory postsynaptic current (mEPSC) amplitude (p) and frequency (q) of neurons in the VTA from WT and SHANK2B^{Y29X} KI mice (P38-40). Neuron number: n(WT)=5, n(KI)=3.

Data are presented as the mean±SEM. Statistical significance was evaluated by unpaired Student’s t-test (c, d, f, g, j, o), Mann Whitney test (p), Wilcoxon test (q) and Two-way ANOVA (a, i, m) followed by Šídák's multiple comparison. *p<0.05, **p<0.01, ***p<0.001.

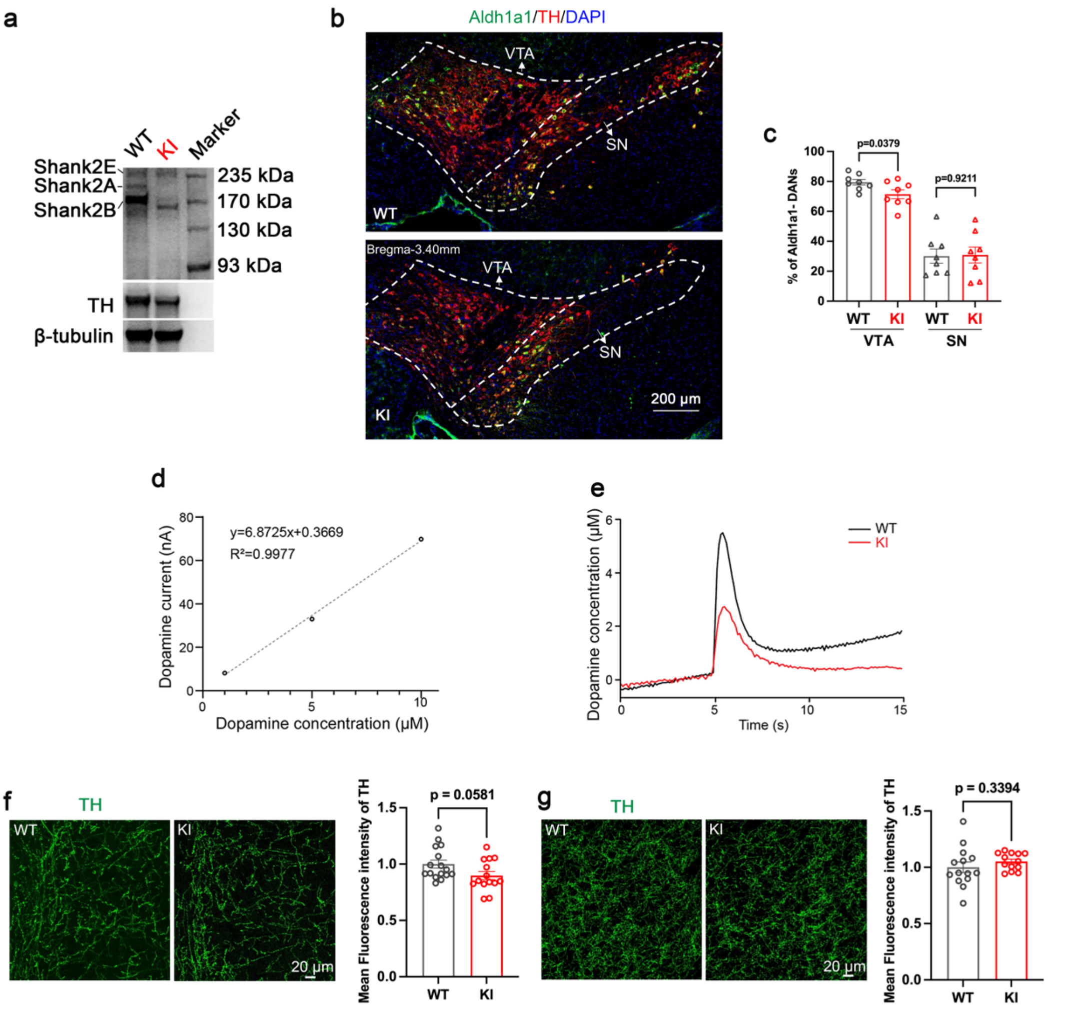

Figure S11. Isoform disruption and developmental defects of dopamine neuron in SHANK2B^{Y29X} KI mice.

a, Western blot of Shank2 (antibody recognizes C terminus of Shank2 protein, antibody from SYSY) and TH in VTA tissue of WT and SHANK2B^{Y29X} KI mice, demonstrating deletion of Shank2B isoforms. GAPDH serves as the loading control.

b, Immunostaining of Aldh1a1 (green) and TH (red) in VTA and SN regions of WT and SHANK2B^{Y29X} KI mice. Dashed lines and arrows indicate SN and VTA in brain sections (bregma: -3.40mm). Scale bar=200 μm.

c, Quantitative analysis of percentage of Aldh1a1 negative dopamine neuron in VTA and SN (n=8 half brain slices from 3 WT mice and 3 KI mice).

d, Calibration curve for the dopamine release concentration measurements. The current peaks (y axis) were recorded by microelectrode at known dopamine release concentration (x axis) to generate calibration curve.

e, Current changes in nucleus accumbens (NAc) evoked by electrical stimulation in VTA region, the concentration and temporal dynamics of dopamine transmitter release can be determined by the dopamine current when comparing to the calibration curve.

f, Immunostaining of TH (green) and corresponding mean fluorescence intensity of TH in medial prefrontal cortex (mPFC) of WT and SHANK2B^{Y29X} KI mice. (n⩾10 image fields from 3 WT mice and 3 KI mice, mean fluorescence intensity was normalized to the average of WT group). Scale bar=20 μm.

g, Immunostaining of TH (green) and corresponding mean fluorescence intensity of TH in basolateral amygdala (BLA) (bregma: -3.40mm) of WT and SHANK2B^{Y29X} KI mice. (n⩾10 image fields from 3 WT mice and 3 KI mice, mean fluorescence intensity was normalized to the average of WT group). Scale bar=20 μm.

Data are presented as the mean±SEM. Statistical significance was evaluated by unpaired Student's t-test.

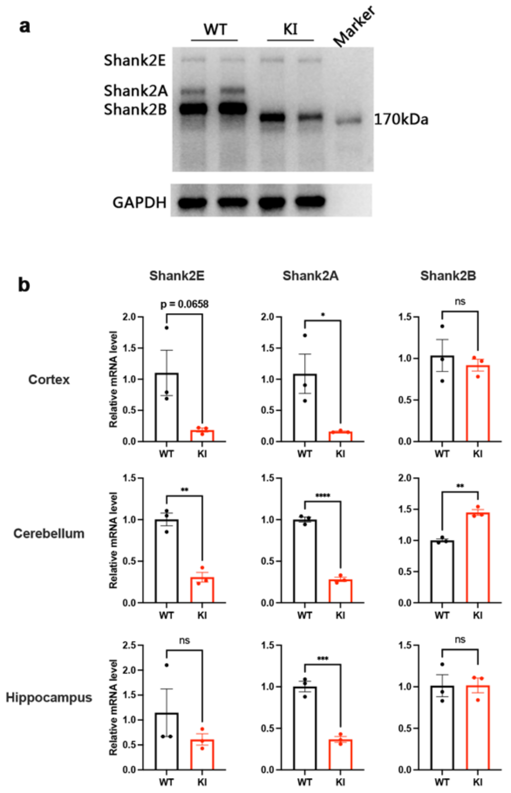

Figure S12. Distinct effects of SHANK2B^{Y29X} mutation on different Shank2 transcripts in mice.

a, Western blot of Shank2 in cortex tissue of WT and SHANK2B^{Y29X} KI mice (antibody from SYSY), demonstrating unchanged Shank2E isoform, lost Shank2A and Shank2B isoform along with a novel short isoform (around 170kDa) in KI mice. GAPDH serves as the loading control. n=2.

b, Relative mRNA level of Shank2E, Shank2A and Shank2B normalized to GAPDH in different brain regions from WT and SHANK2B^{Y29X} KI mice. n=3.

*$p<0.05$, **$p<0.01$, ***$p<0.001$, ****$p<0.0001$, ns: not significant. Unpaired Student's t-test.

a

b

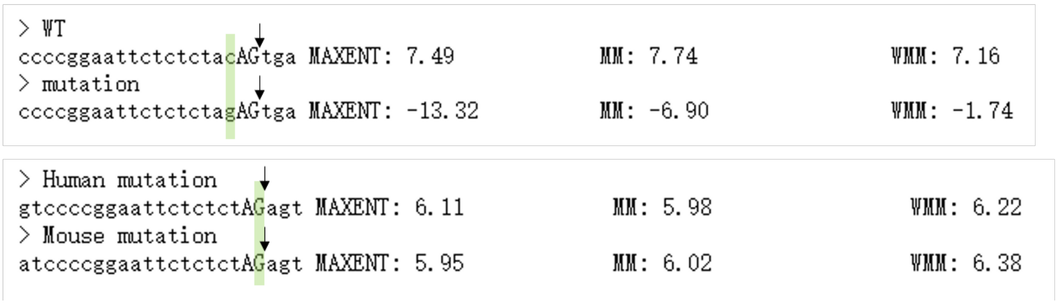

Figure S13. Results output from MaxEntScan show the possibility of splicing event occurring at two different 3' splicing (AG) site with or without *SHANK2* c.87C>G mutation in both human and mouse (top panel). Bottom panel displays the results obtained from a 2bp shift upstream at the 3' splicing site with the SHANK2 c.87C>G mutation in human or mouse. Higher scores indicate a higher probability of the sequence being recognized and spliced at the indicated site by the spliceosome machinery. Black arrow: indicated splicing site for prediction. Green highlighted: *SHANK2* c.87C>G mutation.

Donor site predictions for human_WT :

Start	End	Score	Exon Intron
7	21	0.71	aaggaaggtcagggg
186	200	0.76	tggagtggtgagaag
2079	2093	1.00	gccaaaggtaagagc
3847	3861	0.94	gattgaggtagggac

Donor site predictions for human_mutation :

Start	End	Score	Exon Intron
7	21	0.71	aaggaaggtcagggg
186	200	0.76	tggagtggtgagaag
2079	2093	1.00	gccaaaggtaagagc
3847	3861	0.94	gattgaggtagggac

Correct 5’ splice site

Acceptor site predictions for human_WT :

Start	End	Score	Intron Exon
280	320	0.80	cgctgctcgtcccttgtccaggatactgagccctgtcatcc
329	369	0.99	cccgtccctcctcctccccaggggcccccaatccggggtgg
676	716	0.97	cttccccgctcccctccacagaggccttcccagctccccac
776	816	0.98	actccccttctgcccccgcaggccacccagaaggtgggggt
1982	2022	0.71	tccccggaattctctctacagtgactgcattattgaggaga
2318	2358	0.96	aatgtttttaattttctgtagatgatgttgttctgaatggg
3382	3422	0.72	gaggaacttttccatttttagaaccgaaaatgtttagtcac
3708	3748	0.82	aggtgctgcttgtcttcccagctgacacacccattgaagaa

Acceptor site predictions for human_mutation :

Start	End	Score	Intron Exon
280	320	0.80	cgctgctcgtcccttgtccaggatactgagccctgtcatcc
329	369	0.99	cccgtccctcctcctccccaggggcccccaatccggggtgg
676	716	0.97	cttccccgctcccctccacagaggccttcccagctccccac
776	816	0.98	actccccttctgcccccgcaggccacccagaaggtgggggt
1980	2020	0.89	tgtccccggaattctctctagagtgactgcattattgagga
2318	2358	0.96	aatgtttttaattttctgtagatgatgttgttctgaatggg
3382	3422	0.72	gaggaacttttccatttttagaaccgaaaatgtttagtcac
3708	3748	0.82	aggtgctgcttgtcttcccagctgacacacccattgaagaa

Early splicing

Correct 3’ splice site

Donor site predictions for mouse_WT

Start	End	Score	Exon Intron
144	158	0.70	gtgatgggtaactgt
241	255	0.98	tttccaggtgtgtct
330	344	0.79	aaacattgtaagatt
445	459	0.68	tagactggttagaag
1049	1063	0.99	caaagaggtgagccc
2079	2093	1.00	gcaaaaggtgagtgt
3423	3437	0.91	tgtgcatgtgagtaa
3708	3722	0.76	gattgaggtagggca

Donor site predictions for mouse_KI

Start	End	Score	Exon Intron
144	158	0.70	gtgatgggtaactgt
241	255	0.98	tttccaggtgtgtct
330	344	0.79	aaacattgtaagatt
445	459	0.68	tagactggttagaag
1049	1063	0.99	caaagaggtgagccc
2079	2093	1.00	gcaaaaggtgagtgt
3423	3437	0.91	tgtgcatgtgagtaa
3708	3722	0.76	gattgaggtagggca

Correct 5’ splice site

Acceptor site predictions for mouse_WT

Start	End	Score	Intron Exon
74	114	0.89	ctctgttacctccccccatagcaaattcccagaacacagat
227	267	0.98	tctgtcctcacctgtttccaggtgtgtctaaccagcagacc
459	499	0.83	ggggctttcctgctcctacagctccagggtgccctgttcta
497	537	0.90	ctagttgactgctatctgcaggccagaccctacctaagtcc
531	571	0.71	taagtcccatgcctcctgtagacccaaagccctagagaact
731	771	0.97	gccctctccctccttctccagccctcaacccccagacttcc
827	867	0.83	cagcactgtctcctgccccagagcagccaccaaaaaacggc
1067	1107	0.91	ggttcttggtcttccttgcagtggggatgctgctgccctcg
1097	1137	0.84	tgctgccctcgctgtcctcagcaaggcatcttcctcaggag
1146	1186	0.85	tgccctccttggctctggtaggactgaaatggcctgagccc
1186	1226	0.90	cctcactccctcacccctcagcgttggcctccatggcgggg
1362	1402	0.78	gctgcacactacattccctaggacgggggagagggagggag
1982	2022	0.77	tccccggaattctctctacagtgactgcattattgaggaca
2240	2280	0.81	ttaacattctgccttcctcagaaaacatctaggcttcctgc
2288	2328	0.92	tttttaattttccatctacagtactgctccaaatgagctgc
2861	2901	0.81	cagagtcttctccacccccagggtacaggtccatgcctgtg
3492	3532	0.98	ctgtgaccctcttcctcccagagtcccgtgggcaggtggga
3569	3609	0.96	agtatttctttgtcttcctagccgatacccccattgaggaa
3882	3922	0.89	gatgttcaagtgttttcgtagctctttgggaagagaaagca

Acceptor site predictions for mouse_KI

Start	End	Score	Intron Exon
74	114	0.89	ctctgttacctccccccatagcaaattcccagaacacagat
227	267	0.98	tctgtcctcacctgtttccaggtgtgtctaaccagcagacc
459	499	0.83	ggggctttcctgctcctacagctccagggtgccctgttcta
497	537	0.90	ctagttgactgctatctgcaggccagaccctacctaagtcc
531	571	0.71	taagtcccatgcctcctgtagacccaaagccctagagaact
731	771	0.97	gccctctccctccttctccagccctcaacccccagacttcc
827	867	0.83	cagcactgtctcctgccccagagcagccaccaaaaaacggc
1067	1107	0.91	ggttcttggtcttccttgcagtggggatgctgctgccctcg
1097	1137	0.84	tgctgccctcgctgtcctcagcaaggcatcttcctcaggag
1146	1186	0.85	tgccctccttggctctggtaggactgaaatggcctgagccc
1186	1226	0.90	cctcactccctcacccctcagcgttggcctccatggcgggg
1362	1402	0.78	gctgcacactacattccctaggacgggggagagggagggag
1980	2020	0.91	tatccccggaattctctctagagtgactgcattattgagga
2240	2280	0.81	ttaacattctgccttcctcagaaaacatctaggcttcctgc
2288	2328	0.92	tttttaattttccatctacagtactgctccaaatgagctgc
2861	2901	0.81	cagagtcttctccacccccagggtacaggtccatgcctgtg
3492	3532	0.98	ctgtgaccctcttcctcccagagtcccgtgggcaggtggga
3569	3609	0.96	agtatttctttgtcttcctagccgatacccccattgaggaa
3882	3922	0.89	gatgttcaagtgttttcgtagctctttgggaagagaaagca

Early splicing

Correct 3’ splice site

Figure S14. Results output from Neural Network indicate aberrant splicing event upon *SHANK2* c.87C>G mutation in both human and mouse sequences. The input sequence is centered on the mutation site, extending 2000bp both upstream and downstream, which contains two 5’ splice sites and two 3’ splice sites. New splicing events (red) are predicted to occur 2bp upstream in both species carrying the mutation, compared to the wild-type sequence.

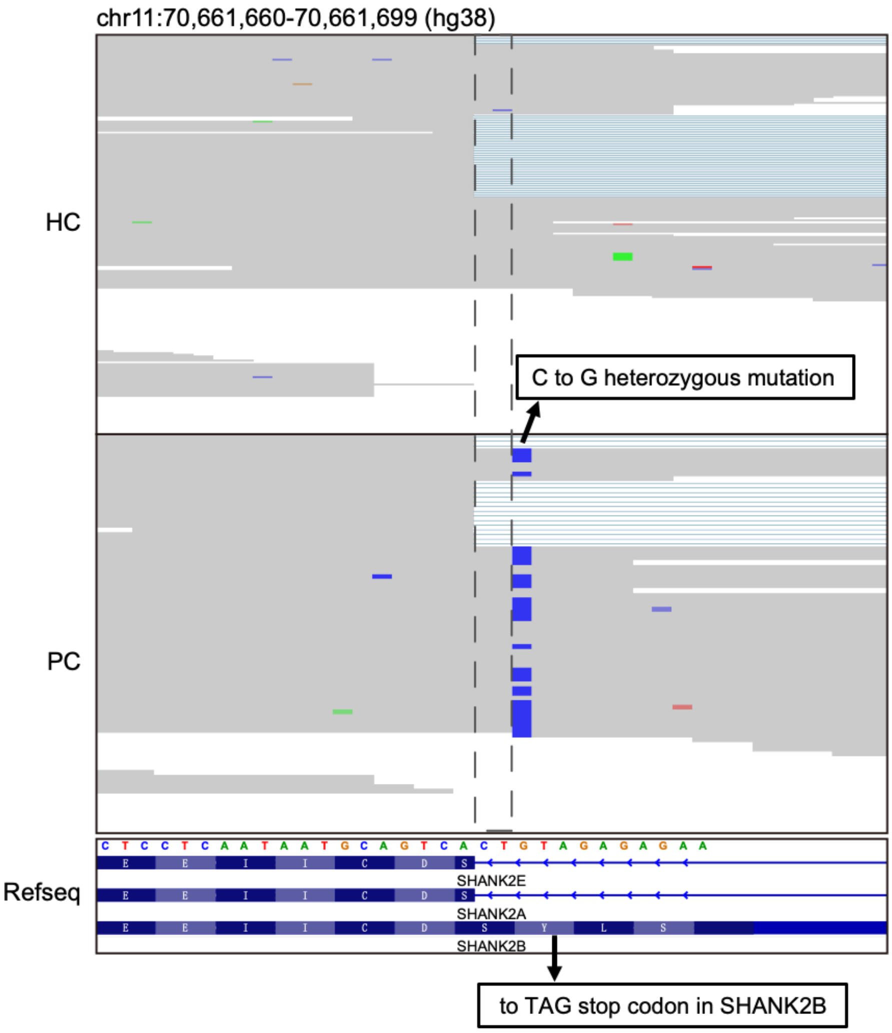

Figure S15. The snapshot illustrates reads obtained from bulk RNA sequencing data of autism patient clone (PC) and his unaffected father (HC) at mutation site. The reads were aligned to the hg38 reference genome and visualized by Integrative Genomics Viewer (IGV).

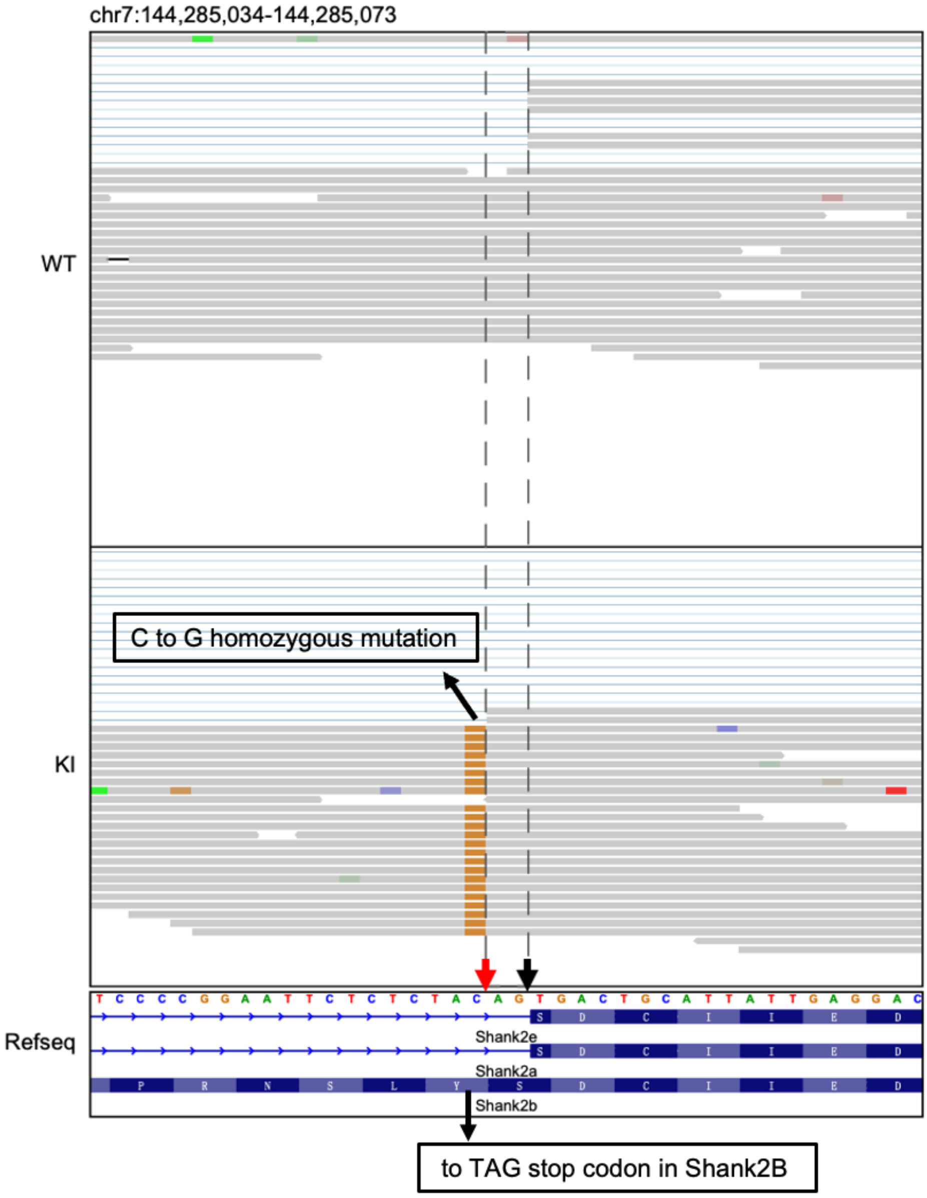

Figure S16. The snapshot illustrates reads obtained from single-cell RNA sequencing data of the E13.5 midbrain in wild-type (WT) and homozygous SHANK2B^{Y29X} KI mice at mutation site. In KI mice, aberrant splicing site (red arrow) was found 2bp earlier than the normal ones (black arrow), resulting in a longer unknown transcript. The reads were aligned to the mm10 reference genome and visualized by IGV.

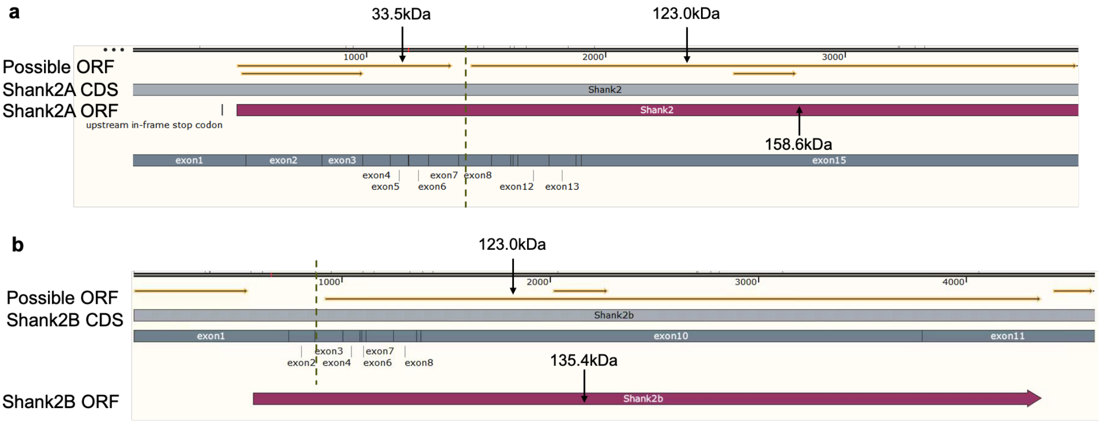

Figure S17. *SHANK2* c.87C>G mutation may create a new open reading frame (ORF) in Shank2 sequences shown by SnapGene v4.1.8.

a, possible ORFs were shown after adding 2bp (AG) within Shank2A transcript.

b, possible ORFs were shown after C>G mutation within Shank2B transcript.

Predicted molecular size of translated proteins are indicated. The 123kDa protein derived from Shank2A or Shank2B might correspond to the novel isoform observed in Western blot. When aligned to the exons of the transcripts, this 123 kDa protein is potentially lacking the functioning PDZ domain, which is generated from exon 4-6 in Shank2A and exon 1-3 in Shank2B.

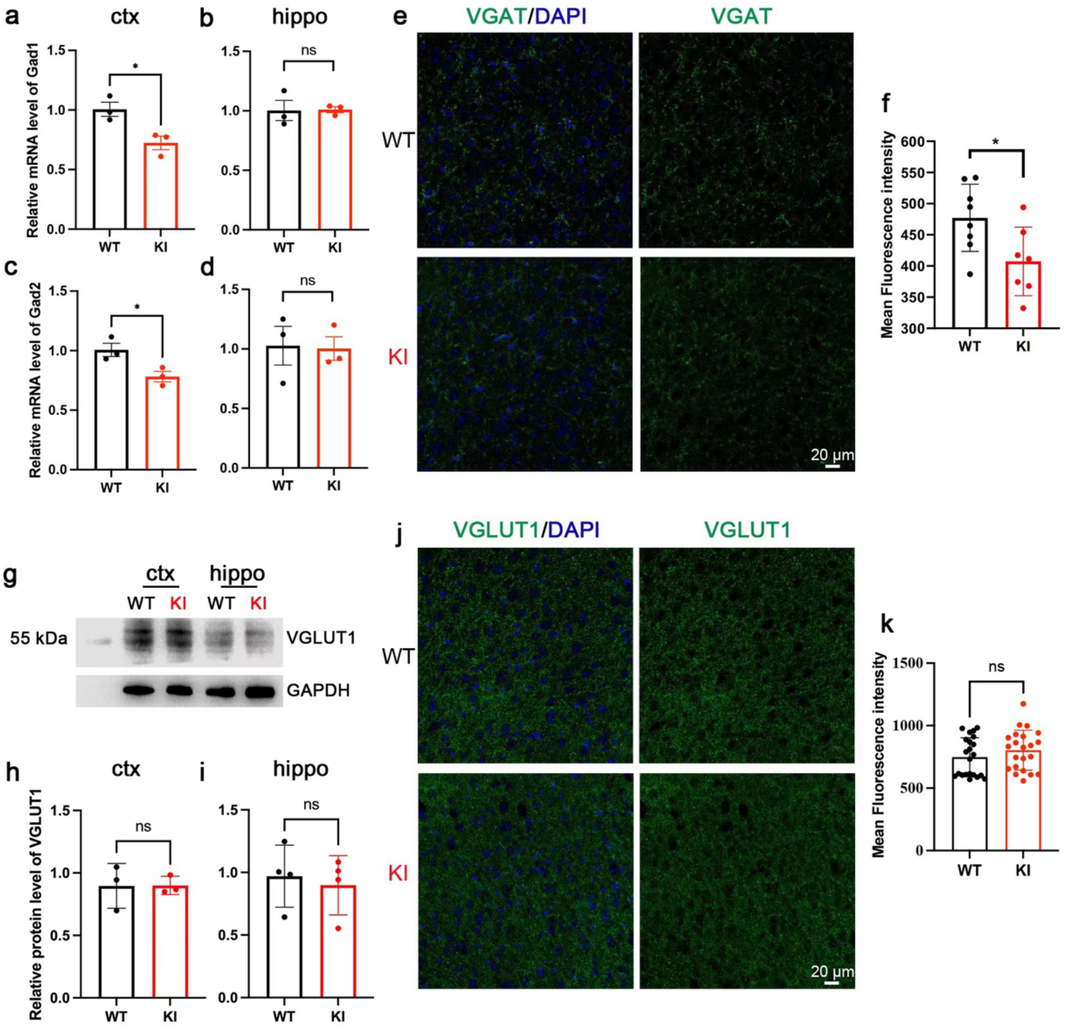

Figure S18. Gross impairments on GABAergic and glutamatergic neurons in SHANK2B^{Y29X} KI mice.

a-d, Relative mRNA level of Gad1 (a, b) and Gad2 (c, d), normalized to GAPDH, in cortex (a, c) and hippocampus (b, d) tissues of WT and SHANK2B^{Y29X} KI mice. (n=3 for each group).

e, Immunostaining of VGAT (green) in prefrontal cortex of WT and SHANK2B^{Y29X} KI mice. Scale bar=20 μm.

f, Quantitative analysis on mean fluorescence intensity of VGAT in WT and SHANK2B^{Y29X} KI mice. Each analyzed dot represents one image field. n=8 image fields from WT and 7 from KI mouse. Data are derived from 1 mouse in each group.

g, Western blot of VGLUT1 in cortex and hippocampus from WT and SHANK2B^{Y29X} KI mice. GAPDH serves as the loading control.

h-i, Densitometric quantification showing the relative protein level of VGLUT1 normalized to GAPDH remains unchanged in both cortex (h) and hippocampus (i) of SHANK2B^{Y29X} KI mice compared with WT mice. (n⩾3 from each group).

j, Immunostaining of VGLUT1 (green) in prefrontal cortex of WT and SHANK2B^{Y29X} KI mice. Scale bar=20 μm.

k, Quantitative analysis on mean fluorescence intensity of VGLUT1 in WT and SHANK2B^{Y29X} KI mice. Each analyzed dot represents one image field. n=22 image fields from WT and 22 from KI mice. Data are derived from 3 mice in each group.

Data are presented as the mean±SEM. Statistical significance was evaluated by unpaired Student's t-test. *p<0.05, **p <0.01, ***p <0.001, ****p<0.0001, ns: not significant.

Table S1. Antibody list.

Antibody	Host species	Concentration used	Reference number	Company
Oct4	Mouse	1:100	6765-100	BD Pharmingen
SSEA4	Mouse	1:100	41-4000	InvitrogenTM
SOX2	Mouse	1:500	MAB2018	R&D Systems
Nestin	Rabbit	1:1000	ABD69	Millipore
Map2	Rabbit	1:500	AB5622	Millipore
Map2	Mouse	1:1000	M9942	Sigma
VGAT	Rabbit	1:500	131013	Synaptic Systems
VGLUT1	Mouse	1:1000	MAB5502	Millipore
TH	Rabbit	1:300	2792	CST
TH	Rabbit	1:1000	AB152	Millipore
Shank2	Rabbit	1:500	162202	Synaptic Systems
Shank2	Rabbit	1:1000	PA5-39454	Thermo Fisher
Anti-GAPDH antibody	Rabbit	1:4000	D110016-0025	BBI
Anti-β actin	Rabbit	1:3000	Ab8227	Abcam
Aldehyde Dehydro genase 1-A1	Goat	10ug/ml	AF5869	R&D Systems
anti-Rabbit Alexa-488	Donkey	1:500	A21206	InvitrogenTM
anti-Mouse Alexa-546	Donkey	1:1000	A10036	InvitrogenTM
anti-Mouse Alexa Fluor 647	Donkey	1:500	A31571	InvitrogenTM
anti-Rabbit IgG (H+L) Peroxidase-conjugated AffiniPure	Goat	1:500	131879	Jackson Immuno Reserch
anti-Rabbit IgG (H+L) Alexa Fluor 488	Goat	1:500	2069632	Invitrogen

anti-Rabbit IgG (H+L) Alexa Fluor 555	Goat	1:500	2018130	Invitrogen
Alexa Fluro 488 conjugate	Streptavidin	1:500	18585036	Invitrogen
Alexa Fluro 555 conjugate	Streptavidin	1:500	1696220	Life technologies

Table S2. Primer list.

Gene/Transcript	forward	reverse
GAPDH	ATCTTCTTGTGCAGTGCCAGC	ACTCCACGACATACTCAGCACC
Human long SHANK2 transcripts (SHANK2E and SHANK2A)	TTGGTGCCAACAAGGACTCA	TATGGCTTGACAGCGACGAA
Human SHANK2B	AATAATGGTCGCTGTCCCCG	TTTTTCTGCAGGACCACCGT
Gad1	AACGTATGATACTTGGTGTGGC	CCAGGCTATTGGTCCTTTGTAAG
Gad2	TCCGGCTTTTGGTCCTTCG	ATGCCGCCCGTGAACTTTT
Mouse Shank2A	GTCTTTGTTAAATGCCTTCAC	GAGGTTGTTGTCACTATTAGAA
Mouse Shank2B	GGTTACAATAATGGTCGCTATC	GGTGTGAATTCCTCAATGGG
Mouse Shank2E	GGTGGAAACAAGGAATTGAAAA	CTTGTGGTTCTTGATGTACTCT
